# Genomic Variation in Rice: Genesis of Highly Polymorphic Linkage Blocks during Domestication

**DOI:** 10.1371/journal.pgen.0020199

**Published:** 2006-11-17

**Authors:** Tian Tang, Jian Lu, Jianzi Huang, Jinghong He, Susan R McCouch, Yang Shen, Zeng Kai, Michael D Purugganan, Suhua Shi, Chung-I Wu

**Affiliations:** 1 State Key Laboratory of Biocontrol, School of Life Sciences, Sun Yat-sen (Zhongshan) University, Guangzhou, People's Republic of China; 2 Department of Ecology and Evolution, University of Chicago, Chicago, Illinois, United States of America; 3 Plant Breeding Department, Cornell University, Ithaca, New York, United States of America; 4 Department of Genetics, North Carolina State University, Raleigh, North Carolina, United States of America; 5 International Center for Evolutionary and Genomic Studies, Sun Yat-sen (Zhongshan) University, Guangzhou, People's Republic of China; University of Wisconsin, United States of America

## Abstract

Genomic regions that are unusually divergent between closely related species or racial groups can be particularly informative about the process of speciation or the operation of natural selection. The two sequenced genomes of cultivated Asian rice, *Oryza sativa,* reveal that at least 6% of the genomes are unusually divergent. Sequencing of ten unlinked loci from the highly divergent regions consistently identified two highly divergent haplotypes with each locus in nearly complete linkage disequilibrium among 25 O. sativa cultivars and 35 lines from six wild species. The existence of two highly divergent haplotypes in high divergence regions in species from all geographical areas (Africa, Asia, and Oceania) was in contrast to the low polymorphism and low linkage disequilibrium that were observed in other parts of the genome, represented by ten reference loci. While several natural processes are likely to contribute to this pattern of genomic variation, domestication may have greatly exaggerated the trend. In this hypothesis, divergent haplotypes that were adapted to different geographical and ecological environments migrated along with humans during the development of domesticated varieties. If true, these high divergence regions of the genome would be enriched for loci that contribute to the enormous range of phenotypic variation observed among domesticated breeds.

## Synopsis

The coexistence of high and low divergence regions in the genomes of two incipient species can be informative about the process of speciation. For example, it may indicate a long period of continual gene flow during species formation. In the conventional view of speciation by geographical separation, there is little intermingling in the process, and the level of divergence should be relatively uniform across the genome. Domesticated plants and animals are excellent materials for studying speciation because the process of domestication may often exaggerate the forces that drive speciation. These forces include selection (artificial rather than natural) and admixture among diverging varieties mediated by humans. In this study, the authors analyzed the whole genome sequences between the two subspecies of domesticated rice. These subspecies have developed partial reproductive isolation. By studying the entire genomic patterns, as well as the detailed population genetics of 20 loci among 60 lines of cultivars and wild rice, the authors observed regions of unusually high divergence, which occupy more than 6% of the whole genome. Hence, the formation of domesticated rice resembles a process in which previously divergent populations/races were brought together, sorted, and re-assembled. How much this process may echo the formation of species in nature is discussed.

## Introduction

Consider two genomes, each from a different population/race of the same species, or from a different but closely related species. In such comparisons, genomic segments that are unusually divergent (between species) or polymorphic (within species), vis-à-vis the genomic average, are of particular interest. These segments can be informative about either the operation of natural selection or the process of race/species formation. For the former, two well-known examples are the major histocompatibility complex and self-incompatibility complexes. Alleles in these systems are often highly divergent and the polymorphisms are maintained by strong balancing selection over long periods of time [1–6]. For the latter, the disparity in the level of divergence among loci has been used as evidence for gene flow during speciation [7–10]. In particular, those loci with a higher level of divergence are more likely to be directly involved in the evolution of reproductive isolation [11,12]. The argument also applies to geographically separated populations, between which strongly differentiated loci are considered candidates for local adaptation and race formation [13,14].

Since the unusual patterns of divergence and polymorphism between genomes provide a convenient window for studying selection and isolation, the search for such cases can be a rewarding exercise. Domesticated animals and plants are promising targets, as domestication may often involve gene admixture across isolation barriers as well as intense selection. The first case where complete genomes from two different domesticated lines have been sequenced is Asian rice, *Oryza sativa.* Genomic sequences from the japonica cultivar Nipponbare (j-Npb) and the indica cultivar 9311 (i-9311) have been published [15,16]. The *indica* and *japonica* sub-species are well differentiated genetically [17–20] and the accumulation of partial sterility barriers helps to maintain their reproductive isolation [19,21].

The AA-genome species complex of rice comprises six wild species, in addition to the two cultivated subspecies. The six wild species, classified by their geographical distribution and life history, are the Asian annual Oryza nivara and perennial *Oryza rufipogon;* the African annual Oryza barthii and perennial *Oryza longistaminata;* the South American form Oryza glumaepatula and the Oceanian annual Oryza meridionalis [19]. O. rufipogon is widely believed to be the major source of rice domestication in Asia [19,22] while recent QTL evidence also supports a possible O. nivara origin of indica rice [23]. Gene flow across the incomplete reproductive barriers separating the six wild and two cultivated species of rice has been well documented [19]. A case in point is the “Obake” plants, which are thought to be derived from introgressive hybridization between O. longistaminata and O. sativa [24], or the recently documented admixtures between the Asian and African cultivated species, O. sativa and Oryza glaberrima [25].

This report provides (i) a survey of the genome-wide divergence pattern between j-Npb and i-9311, and (ii) a comparative summary of the population genetics between the high divergence regions and the rest of the genome. Implications of the observed pattern of divergence for rice domestication and the forces shaping it will be discussed.

## Results

### The Existence of High Divergence Regions between j-Npb and i-9311

**Figure 1:**
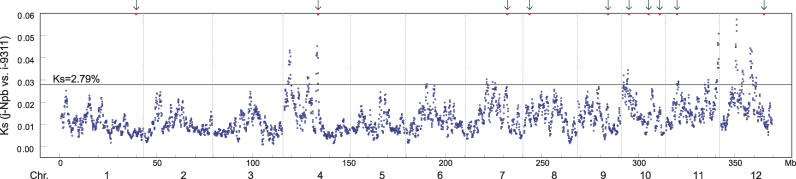
A Sliding Window Presentation of the Mean Ks Values between j-Npb and i-9311 for Genes in Each 1-Mb Segment The step size is 0.1 Mb. Each Ks value is obtained from the concatenated sequences within the segment. The horizontal line is the cutoff at Ks = 2.79% (see text). Arrows on the top indicate the positions of high polymorphism genes used in Table 2. Only half of them were chosen from large clusters of high polymorphism genes (>1 Mb). A parallel presentation that adjusts for variation in mutation rate across the whole genome is given in Figure S2.

In comparing the two genomes of j-Npb and i-9311, we first removed all transposable element-like sequences, retaining 31,023 gene models. Of these, 15,406 have been confirmed by EST sequences [26]. The mean level of divergence (measured in Ks, number of synonymous substitutions per site) among the 31,023 genes is 1.15%. We then used a sliding window approach to display the variation in Ks across the genome (Figure 1). In this display, each 1-megabase (Mb) window contains on average 84 genes. With a step width of 0.1 Mb, there are a total of 3,587 windows covering 369.5 Mb.

**Table 2:**
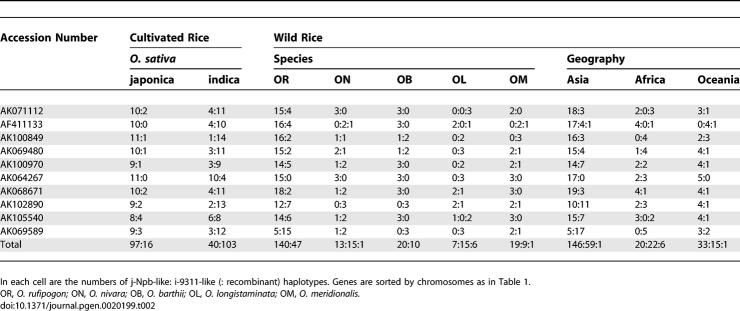
The Distribution of Haplotypes for High Polymorphism Genes by Species or Geography

**Table 1:**
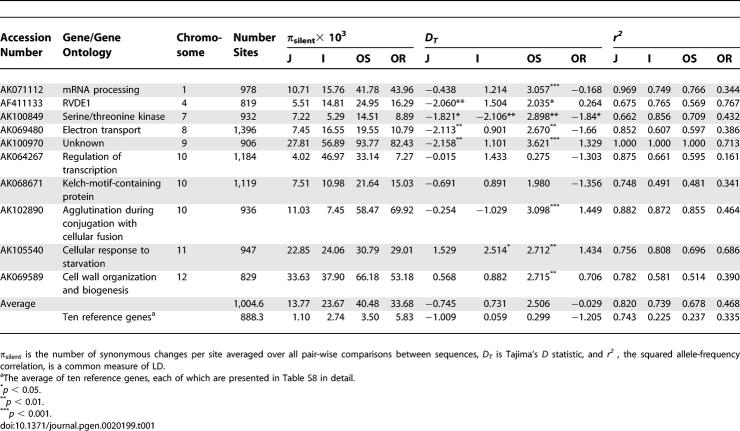
Statistics for High Polymorphism and Reference Genes in Rice Cultivars and O. rufipogon

A glance at Figure 1 suggests that there are several peaks where Ks values are significantly higher than background. To see whether these peaks are biologically meaningful, we selected ten loci from the high divergence regions and ten more from regions of average variation for further sequencing among cultivars and wild rice (Table S1; see next section).

In this section, we asked whether genes of high divergence were significantly clustered and, if so, whether some genomic factors, such as mutation rate, might account for the clustering of high divergence genes. Genes and intergenic segments were randomly shuffled 1,000 times (see Materials and Methods). For each permutation, the pseudo-genome was subjected to the same sliding window analysis, and the highest Ks value (Kmax) among the 3,587 1-Mb segments was recorded. We used Kmax to determine the significance of each observation. Among the 1,000 Kmax values, ten have a Kmax > 2.79%, which was used as the cutoff for defining high divergence regions (*p* < 0.01). In Figure S1, our study using the coalescence approach showed that the cutoff at 2.8% indeed exceeds the simulated divergence values for all 1-Mb segments in the rice genomes. The distribution of such divergence values is very sensitive to even a small amount of recombination, which was calibrated using the entire “mismatch distribution” (see legends of Figure S1 for detail).

In Figure 1, the horizontal line is the cutoff at Ks = 2.79%. Thus, the probability of observing even one single 1-Mb segment that rises above this cutoff, among the 3,587 segments in each permutation, is *p* < 0.01. In Figure 1, there are 14 segments that rise above this threshold with an average length of 1.56 Mb. In total, the high divergence regions account for 5.9% of the genome. (Note that the number of segments with Ks > 2% in each random permutation is generally much smaller than the observed; hence, the cutoff at 2.79% may be conservative for estimating either the number or the size of the high divergence regions.)

We first ask if local variation in mutation rate may account for the presence of the high divergence regions. To calibrate the effect of mutation-rate variation, we used genomic sequence available from bacterial artificial chromosome end sequencing [27] of a wild rice species with an FF genome, *Oryza brachyantha,* as an out-group. Divergence between *O. sativa* and the out-group is almost uniform across the entire genome, such that dividing the divergence level of Figure 1 with the distance to the out-group yields a nearly identical pattern (see Figure S2). We thus rule out higher mutation rate as an explanation for the existence of these high divergence regions, each over 1 Mb long.

We then asked if other genomic features, such as GC content, transposable element (TE) abundance, large chromosomal inversions, and proximity to centromeres might be strongly correlated with the level of divergence. If they do, we may seek explanations for these features and, indirectly, the existence of the high divergence regions. As shown in Table S2, both GC content (44% versus 43%) and TE abundance (47.2 versus 48.2 elements per Mb) are very similar between the two regions. The correlation between Ks and either genomic feature is also very weak, with less than 2% of the variation in Ks explained.

Neither are chromosomal features likely to be significant factors. Large chromosomal inversions, if present, might prevent recombinations between haplotypes of j-Npb and i-9311. As has been done in Lu et al. (2006) [28], we found that the correlation between the level of nucleotide diversity and local recombination is low (*r^2^* = 0.025, *p* > 0.05). Previous genetic experiments between japonica and indica [29] have also suggested that cultivars do not harbor large inversions. Comparative genomic analysis between j-Npb and i-9311 [30] are not informative about large inversions, and putative small inversions do not appear to be distributed differently between high and normal divergence regions (see Table S3). Furthermore, the high divergence regions are often a distance away from the centromere (on average about 5.1 Mb away) and only one such region straddles a centromere (on Chromosome 12). In short, the contrast between high and low divergence regions has to be explained by factors other than genomic or chromosomal features.

In Tables S4–S6, we compiled lists of genes that are overrepresented in the high divergence regions. By the “biological process” classification according to Gene Ontology (GO), genes in the following categories are overrepresented: response to biotic stimulus, signal transduction, protein and macromolecule metabolism, and flower development (Table S4). By “molecular function,” genes in various binding classes and transferase activity genes are overrepresented (Table S5) while cell wall genes are most abundant by the “cellular component” assignment (Table S6). These categories hint traits of agricultural relevance including biotic stress and signal transduction. Genes known to be associated with rice domestication are not preferentially clustered in regions of high divergence (Table S7). This is not surprising because most of these genes distinguish O. sativa from the wild progenitors, whereas our search was for genes that separate the indica and japonica subspecies within O. sativa.

### Population Genetics of High Divergence Regions among Cultivars and Wild Rice

To understand the nature of the contrasting levels of sequence divergence in different genomic regions as illustrated in Figure 1, we surveyed the variation among 25 lines of O. sativa consisting of 11 japonica-like and 14 indica-like accessions, 20 lines of *O. rufipogon,* and three lines from each of five other AA-genome wild species (Table S1). Both landrace and elite varieties were included in the collection of O. sativa (see Table S1). The j-Npb and i-9311 sequences were included in the analyses. Most of our japonica-like lines are from the temperate zone of China and most of the indica-like lines are from southeastern or southern Asia. As will become clear later, the geographical distribution of these lines is most germane to our observations; hence, the “indica-like” or “japonica-like” designation is used to reflect that emphasis. The collection of O. rufipogon came from the known distribution of this species, including its most northern distribution in China (Jiangxi Province, see Table S1). O. rufipogon lines in China were collected from wild populations (see Table S1).

For sequencing, we selected ten genes with Ks > 5% between j-Npb and i-9311 to represent the high divergence genomic regions. The positions of the ten high divergence genes are indicated with arrows in Figure 1. Note that the peaks in Figure 1 represent contiguous regions (>1 Mb) of high divergence genes. There are also many small islands of high divergence that would not be visible in this figure. The loci chosen are distributed equally between large (>1 Mb) and small (<1 Mb) islands of high divergence. For reference genes, we chose ten well-characterized genes from the rest of the genome; all genes used in this analysis have corresponding full-length cDNA sequences. About 1 kb of each gene was sequenced in the 60 accessions.

For the purposes of this analysis, we used only single copy genes believed to be orthologous in cultivated and wild species. To select genes that conformed to this rule, we performed extensive BLAST searches against the available j-Npb and i-9311 genomic sequences to rule out multiple copy gene sequences. For each of the ten high divergence genes chosen for re-sequencing, we also carried out Southern analysis using genomic DNA of j-Npb, i-9311, and O. rufipogon digested with two different restriction enzymes (see Figure S3). In each case, only a single hybridizing restriction fragment was observed. From these lines of evidence, we concluded that the re-sequenced genes from the ten high divergence regions, as well as those from the reference regions, could be considered single-copy orthologs in *Oryza.*

The synonymous nucleotide diversity in O. sativa and O. rufipogon was compared between the two categories of genes. High divergence genes, on average, had about 10-fold higher levels of diversity than the ten reference genes (Table 1). This was true for both O. sativa and *O. rufipogon.* On a genome-wide basis, domesticated crops and animals tend to have reduced genetic diversities relative to their wild progenitors [31]. Both population bottlenecks and strong selection contribute to the reduction [32–35]. Averaged over the ten reference genes, there is indeed substantial reduction in the silent nucleotide diversity among rice cultivars (indicas, japonicas, or all cultivars combined, with π_silent_ = 1.1 − 3.5 × 10^−3^, bottom row of Table 1) when compared with O. rufipogon (π_silent_= 5.83 × 10^−3^).

The high polymorphism genes, however, behave differently. (Note that high divergence refers to the comparison between i-9311 and j-Npb. When multiple lines are analyzed, we refer to the same phenomenon as high polymorphism.) The mean silent diversity among all cultivars is in fact somewhat higher than that in O. rufipogon (π_silent_= 40.48 × 10^−3^ versus 33.68 × 10^−3^); this pattern is observable in eight of the ten high divergence genes. The patterns documented in Table 1 suggest that the different sub-populations of O. sativa captured different portions of the genetic diversity of the ancestral O. rufipogon population, and, in combination, preserved much of the diversity observed in the high polymorphism regions of the genome.

Tajima's *D* [36] is more positive (or less negative) for high polymorphism genes in both cultivated and wild rice (Table 1). This trend indicates that there is a greater representation of intermediate frequency alleles in high polymorphism genes than in reference genes. Most notable is the very high positive *D* (2.506) in the combined *indica* and *japonica* samples of O. sativa among high polymorphism genes (Table 1). This trend reflects the strong differentiation between the *indica* and *japonica* cultivar groups (see below).

### Persistence of Two Highly Divergent Haplotypes among Many Species

**Figure 2:**
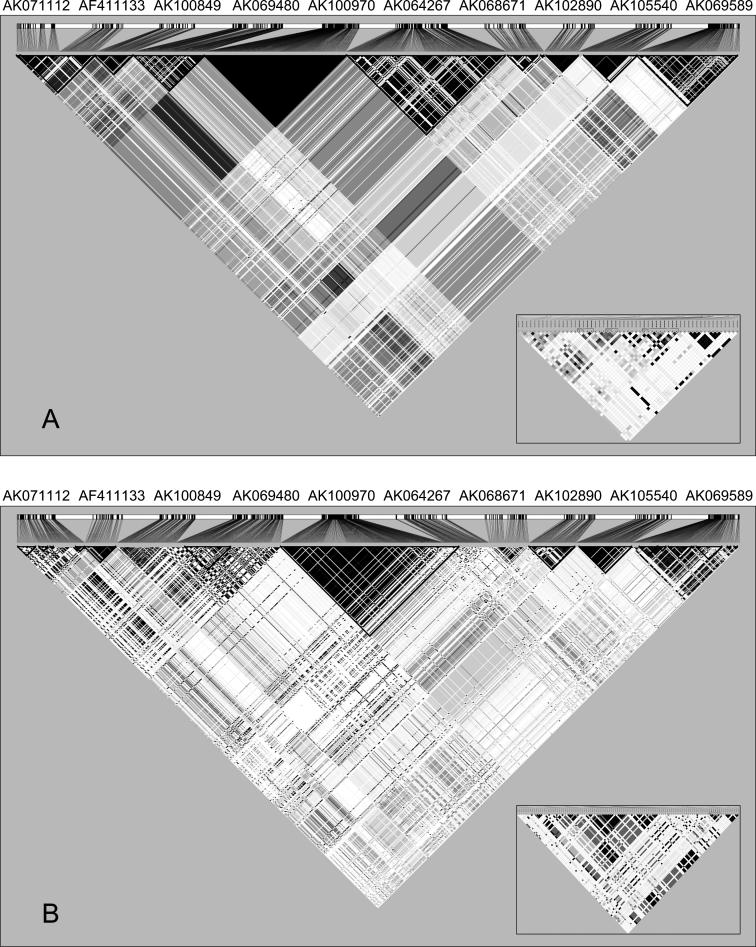
LD Patterns for High Polymorphism and Reference Genes LD are shown by the *r^2^* statistic, with white for *r^2^* = 0, shades of gray for 0 < *r^2^* < 1, and black for *r^2^* = 1. Genes are arranged by chromosome as in Figure 1 and Table 1. The haplotype blocks are delineated by bold lines. (A) *O. sativa.* (B) O. rufipogon. In both panels, the main one is for high polymorphism genes and the inset is for the less polymorphic reference genes.

The high divergence genes are not only highly variable at the nucleotide level; they are also in strong linkage disequilibrium (LD) as shown in Figure 2A and 2B. This is true for both O. sativa and O. rufipogon (see Table 1 for the *r^2^* values). The trend is even stronger if we consider the *indica* and *japonica* sub-groups separately. At the same time, LD decays more rapidly in regions of normal polymorphism than in regions of high polymorphism in O. sativa (Mann-Whitney U test, *p* < 0.001 for *r^2^* values; see also insets of Figure 2A.) The contrast in LD between regions of high and low polymorphism is not as pronounced in O. rufipogon as in O. sativa mainly because LD in the high polymorphism regions in the outcrossing O. rufipogon is not as high.

In theory, highly polymorphic regions are expected to be older, and, having more time to recombine, should have a lower level of LD. Because the observation is opposite to this expectation, the high LD for the high polymorphism genes suggests either (i) balancing selection, or (ii) recent admixture with insufficient time for LD decay. In rice cultivars, selfing should further retard the decay of LD after admixture and, indeed, even unlinked high polymorphism genes show some degree of LD.

**Figure 3:**
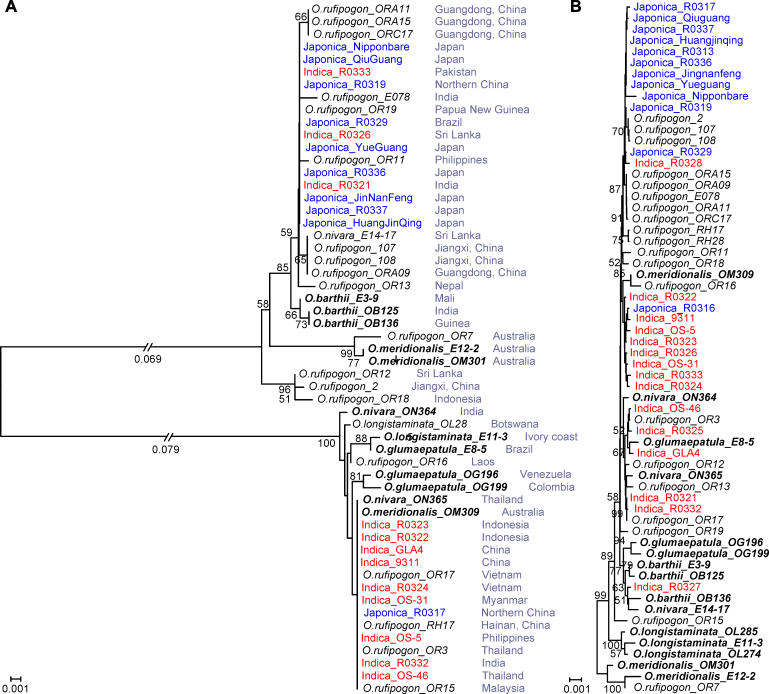
Contrasting the Phylogenies for High Polymorphism and Reference Genes (A) Neighbor-joining tree of a high polymorphism gene *AK100970* with the geographical origins of the samples noted. (B) Neighbor-joining tree of the ten reference genes listed in Table S8. The bootstrap values are indicated at nodes with at least 50% support. Cultivars are labeled in blue for japonicas and in red for indicas. O. rufipogon are indicated by italics and other wild species are indicated by boldface. Note that the size of the tree shown in Figure 3A is only half of the actual height.

**Figure 4:**
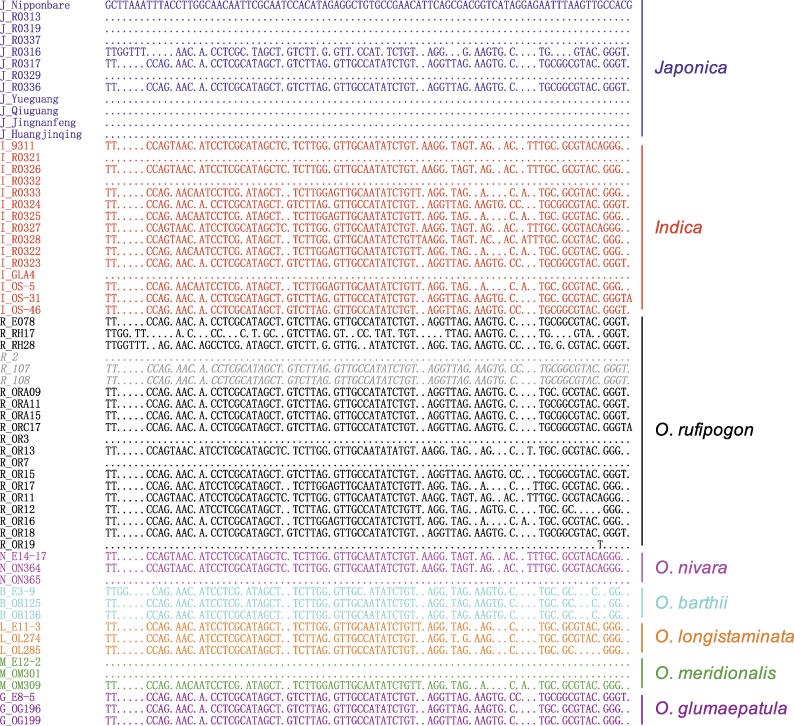
An Example of Haplotypes from a High Polymorphism Gene *AK069589* Haplotypes were identified by the polymorphic sites in *O. sativa.* There are two distinct haplotypes that differ from each other by nearly 100 substitutions. Both subspecies of cultivars and the three wild species, *O. rufipogon, O. nivara,* and *O. meridionalis,* carry both haplotypes. The two haplotypes also coexist in the same population of O. rufipogon from Jiangxi, China, the three specimens of which are indicated in gray.

With high polymorphism and strong LD, it is expected that the haplotypes would be partitioned into deeply divided clades. Figure 3A illustrates this for one gene and the rest are given in Figure S4. For comparison, the phylogeny based on the ten reference genes is given in Figure 3B. This latter phylogeny is congruent with the known history of rice cultivation [37] as all rice cultivars appear to be derived from the Asian wild rice, *O. rufipogon,* and the Australian O. meridonalis is the most distantly related species (Figure 3B). The phylogenies of high polymorphism genes in Figures 3A and S4 are all much more divergent than that of Figure 3B. (Note that the scale in Figure S4 is five to 20 times greater than that of Figure 3B). Yet, it should be noted that all these phylogenies show a deep division between the j-Npb-like and i-9311-like haplotypes. For some genes like *AK069589,* the two haplotypes differ by nearly 100 substitutions (Figure 4), in contrast with the reference genes, which usually have only a few polymorphic sites.

Most intriguing, the divergence of these haplotypes is older than the species divergence. For example, in Figure 3A, all cultivars as well as the three wild species, *O. rufipogon, O. nivara,* and O. meridionalis all have lines on each side of the deep divide. For gene *AK069589,* the two haplotypes also coexist in the same population of O. rufipogon from Jiangxi, China (Figure 4). In Table 2, we show the distribution of the two types of haplotypes for each locus among species, as well as among geographical areas. The two distinct haplotypes and the occasional recombinants are easily recognizable based on an inspection of the DNA sequences (see Figure 4). In Table 2, we can see that the presence of the two haplotypes in the extant species is not restricted to a couple of species or defined geographical areas. Most species and geographical areas harbor both haplotypes at most loci.

## Discussion

*Oryza* accessions collected from diverse geographical areas share groups of genes that comprise two highly divergent haplotypes in strong LD. These polymorphisms are older than the AA genome rice species sampled in this study. How have such large LD blocks of high polymorphisms been maintained and what may be the significance of such regions?

In the Introduction we reviewed current explanations for the existence of highly divergent regions in the genome; namely, balancing selection or continual gene flow during incipient speciation. The simplest form of balancing selection is over-dominant selection, a phenomenon that underlies the polymorphisms of MHC in animals [1–3], and self-incompatibility, which is common in plant systems [4–6]. Loci associated with over-dominant selection usually occupy defined and relatively small portions of the genome [38,39], but the regions of high polymorphism in rice are exceptionally large, spanning perhaps a Mb (see Figure 1). It is therefore doubtful that over-dominant selection is the cause of such extensive polymorphisms.

Another possible explanation is admixture among species in the recent past. If each of the different *Oryza* species originally contained a distinct haplotype, these haplotypes could have been brought into contact recently (perhaps by humans). Indeed, *O. longistaminata, O. meridonalis,* and other wild species often carry a different haplotype from one another (Table 2). The strength of this “species admixture” hypothesis is that it requires no mechanism to explain the existence of high polymorphism, which is simply the result of recent admixture between different species.

In rice, the wild AA-genome species are not “good species” in the sense that they do not experience complete reproductive isolation from one another. Historically, researchers considered them all to be members of a single complex species known as Oryza perennis [19]. In fact, Figure 3B shows that the average divergence among wild AA-genome species is only slightly larger than that among O. sativa cultivars. Gene flow across geographically isolated populations could be a factor in reducing divergence. Second, the two divergent haplotypes are observable in all species tested and both haplotypes are distributed over wide geographical areas. The two divergent haplotypes may represent adaptations to different climates or ecologies and may have existed at very different frequencies in different regions in the past. The two haplotypes may have become intermingled, possibly as a result of human migration coupled with evolving agricultural practices. Under this hypothesis of geographical differentiation and recent admixture, some genomic regions are expected to be much more polymorphic than others [11,12]. The sizes of these regions and the strength of LD depend on the degree of prior geographical differentiation, the extent of local adaptation, corresponding strength of selection, and the timing of admixture before present.

The geographical admixture hypothesis is consistent with two additional observations. First, the *indica* and *japonica* subspecies, which are believed to result from independent domestication events occurring in different geographic regions of the world [22,40], show very different compositions of the two haplotypes. Table 2 shows the relative abundance of the j-Npb: i-9311 haplotype among all 25 *indica*-like and *japonica*-like lines. Among all loci, the ratio is 97:16 for the *japonicas* and 40:103 for the *indicas* (*p* < 10^−9^, the *x^2^* test).

Second, O. rufipogon populations also show a relatively high degree of LD. Considering that the selfing O. sativa lines have an average *r^2^* of 0.237 in regions of normal polymorphism (see Table 1), one may find the level of LD in the outcrossing O. rufipogon (at *r^2^* = 0.468 for high polymorphism genes) somewhat unexpected. LD in the latter should have been eroded a long time ago. Hence, the unexpected LD in O. rufipogon may suggest population sub-structure in this wild ancestor that parallels that of *O. sativa.* This is in keeping with the independent domestication events associated with *indica* and *japonica.* Further, it suggests that the regions of high divergence may be enriched with genes of biological interest. They may include (i) genes that are associated with the numerous sterility barriers that help to maintain the genetic isolation of the two sub-species or populations, and (ii) genes that confer an adapatative advantage to specific ecological conditions and geographical regions. Tables S4–S6 offer some evidence of the latter category. In summary, O. sativa and its immediate progenitor, *O. rufipogon,* as well as the AA-genome complex known as O. perennis show signs of admixture between previously divergent populations. However, it is not clear whether human activity is responsible for the episodes of hybridization between these divergent populations or whether natural zones of hybridization were discovered and exploited by humans during the process of rice domestication.

The enormous phenotypic variation observed in most domesticated plants and animals poses an intriguing question about the source of the underlying genetic diversity. Variation in the wild may have been maintained across a large geographical area. Indeed, the two subspecies of domesticated rice are believed to have derived their genetic variation from different geographical populations of O. rufipogon [17,22,25] (see also Table 2). During domestication, humans appear to have exploited the available genetic diversity from numerous geographical sources. This pattern can be more clearly observed in regions of high divergence than in less differentiated genomic regions. Importantly, some of the genes that reside in these regions of high polymorphism are likely to correspond to loci of biological significance. These loci may control traits associated with reproductive incompatibility or ecological adaptation, as has been inferred for natural systems [11,12]. The sequencing of other domesticated species [41–43] should make it possible to identify regions of unusual divergence and to understand the relationship between these regions and phenotypic traits of interest to evolutionists, biologists, and plant breeders.

## Materials and Methods

### Plant materials.

We sampled 25 lines of the domesticated rice (including 11 japonica and 14 indica cultivars), 20 lines of O. rufipogon from Asia and Oceania, and three lines each from the remaining wild rice in the AA genome complex (i.e., *O. meridionalis, O. barthii, O. nivara, O. glumaepatula,* and O. longistaminata). Detailed information is listed in Table S1 and its legends.

### Analysis of sequence data from j-Npb, i-9311, and *O. brachyantha.*

The genomic sequences of i-9311 with 4× coverage were downloaded from http://btn.genomics.org.cn:8080/rice. Shotgun BAC-end sequences of O. brachyantha were downloaded from the National Center for Biotechnology Information (http://www.ncbi.nlm.nih.gov. Accessed 21 January 2005). These sequences were aligned against the j-Npb genome (assembled in TIGR rice genome pseudomolecule release 3.0). After removing all the transposable element-like genes, we retained 31,023 orthologous genes between j-Npb and i-9311 (15,406 of them confirmed by EST sequences) and 7,588 orthologous genes between j-Npb and O. brachyantha (4,127 of them confirmed by EST sequences). The details about sequence quality control, sequence alignments, annotation extraction, EST matching, and GO were described in Lu et al. [28]. The alignments and analyses are also presented on the Web site: http://pondside.uchicago.edu/wulab.

To observe the variation from region to region, we performed a sliding window analysis with the window size of 1 Mb and the step size 0.1 Mb for each of the 12 chromosomes. A total of 3,587 sliding windows were obtained across the rice genome of 369.5 Mb. For each window, we concatenated all the genes and used the method of Li [44] to compute the “weighted” mean Ks value in each window (Figure 1). On average, each segment contains 84 genes. To control for the stochastic and demographic effects, we shuffled the genome randomly in units of genes/ intergenic regions with 1,000 permutations. The length of each gene or intergenic region was obtained according to TIGR rice pseudomolecule release 3.0. After shuffling, we performed the same approaches of sliding window analysis and calculations of Ks for each pseudogenome as described above. The highest Ks value (Kmax) among the 3,600 or so 1-Mb segments was recorded to determine the significance of the observation. Among the 1,000 Kmax values, 10 have a Kmax > 2.79%, which is set as the cutoff for the high divergence regions with *p* = 0.01.

To exclude the possible effect of mutational heterogeneity across the genome, we calibrate the mean Ks value between j-Npb and i-9311 against that between j-Npb and *O. brachyantha* and present the result in Figure S2. The general patterns in Figures 1 and S2 are very similar, and these patterns do not change whether we used all genes or only genes confirmed by EST sequences. To assess the correlation between levels of Ks and genomic features, we calculated GC content and TE abundance by using a 1-Mb window moving along each of the 12 chromosomes with 0.1-Mb intervals. TIGR *Oryza* Repeats V. 3.1 were submitted to BLAST search against TIGR rice pseudomolecule release 3.0, to determine the positions of each TE superfamily along the entire set of chromosomes. We then verified TE positions manually to eliminate redundancies and integrate nested insertions. We also counted the number of orthologous genes with different orientation between j-Npb and i-9311 genomes against the total number of orthologous genes in a given region, which were used as a measure of inversion abundance. Fisher's exact tests were conducted to compare the abundance of inversions between high divergence regions and the genome for each chromosome and the entire, respectively. Positions of high divergence regions were compared with the centromere locations retrieved from http://www.tigr.org/tdb/e2k1/osa1/pseudomolecules/centromere.shtml.

Genes in high divergence regions were tested for overrepresentation using complete gene annotations as reference and BiNGO [45]. Genes for which there is no annotation are not taken into account in the overrepresentation analysis. Neither were they counted in the test or reference set. Total numbers of tested and reference genes are 398 versus 10,799 (GO Biological Process), 514 versus 14,170 (GO Molecular Function), and 202 versus 5,537 (GO Cellular Component), respectively. Protein coding genes that are related to important phenotypes were downloaded from Gramene Genes Database (http://www.gramene.org/rice_mutant/index.html) and then mapped onto the j-Npb genome.

### Choice of genes and sequencing.

The ten high polymorphism genes were chosen from the whole genome comparison between j-Npb and i-9311 as described above and in Lu et al. [28]. They all have full-length cDNA in Genbank or KOME (http://cdna01.dna.affrc.go.jp/cDNA) and a Ks value of 5% or greater between j-Npb and i-9311. The positions of the ten genes are given at the top of Figure 1. The ten reference genes of Table 1 were chosen only on the basis of functional annotation without prior knowledge of their divergence between japonica and indica. PCR and sequencing reactions are standard procedures [46] and the primers used are available upon request.

### Analysis of sequence data collected in this study.

The sequencing results were assembled using SeqMan (DNASTAR, http://www.dnastar.com). Multiple alignment of sequences was done using Clustal X program [47]. Manual check was performed in every case to ensure sequencing and alignment quality. Sites with alignment gaps were completely excluded in the analysis. Synonymous nucleotide diversity and Tajima's *D*-test were calculated using the program DnaSP version 4.0 [48]. Squared allele-frequency correlations for LD (*r^2^*) were calculated with program SITES [49]. Phylogenetic reconstruction was done with the neighbor-joining method [50] based on Kimura's two-parameter distances [51] and implemented in MEGA version 2.1 [52]. 1,000 bootstrap replications were performed to assess the confidence in the phylogeny. We used polymorphic sites within O. sativa population to identify haplotypes at each high polymorphism gene with the help of DnaSP. LD between pairs of sites was also plotted with *r^2^* scheme as implemented in Haploview version 3.2 [53]. Singletons were included in the LD calculations as there were few segregating sites for most of the reference genes. When singletons are excluded, the LD patterns of high polymorphism genes do not change.

### Southern blot and hybridization of genomic DNA.

The genomic DNA of one individual for each of Nipponbare, 9311, and O. rufipogon were digested with restriction enzymes, which do not cut in the region covered by the probe. The digested genomic DNA was then fractionated by agarose gel electrophoresis and transferred to Hybond-N+ nylon membranes by vacuum pump. The probes were labeled with ^32^P using the Random Primer DNA Labeling Kit (TaKaRa Bio Incorporated, http://www.takara-bio.com). After overnight hybridizations, the nylon membranes were washed according to the AlkPhos Direct protocol (GE Healthcare, http://www.gehealthcare.com) and subjected to autoradiography.

## Supporting Information

Figure S1 Distribution of Neutral Divergence in Ks among All 1-Mb Segments between j-Npb and i-9311 Genomes as the Mismatch Distribution (A) The observed mismatch distribution. (B–D) Simulated distributions under a series of recombination values with 4*Nc*/4*Nμ* = 0, 0.0025, and 0.0125.
(72 KB DOC)

Figure S2 A Sliding Window Presentation of the Mean Ks Value between j-Npb and i-9311 Divided by That between j-Npb and O. brachyantha
The rest is the same as in Figure 1.
(319 KB PPT)

Figure S3 Southern Blot and Hybridization Analysis of Genomic DNA Isolated from Cultivars and Wild Rice Using the Indicated Enzymes and Probes The PCR-amplified fragments were included as positive controls.
(2.2 MB PPT)

Figure S4 Neighbor-Joining Trees among Rice AA-Genome Complex for the Ten High Polymorphism Genes Note that the scale here is five to 20 times greater than that of Figure 3B. The bootstrap values are indicated at nodes with at least 50% support, out of 1,000 replications. Cultivars are labeled in blue for japonicas and in red for indicas. O. rufipogon are indicated by italics and other wild species are indicated by boldface.
(195 KB PPT)

Table S1 Oryza Plant Material Used in This Study (103 KB DOC)

Table S2 Summary of GC Content and TE Abundance by Sliding Window Analysis of 1-Mb Window Length and 0.1-Mb Intervals across j-Npb Genome (36 KB DOC)

Table S3 The Abundance of Inversions between j-Npb and i-9311 in High Divergence Regions and in the Whole Genome High divergence regions are distributed on Chromosomes 4, 6, 7, 10, 11, and 12. Fisher's exact tests were conducted to compare the abundance of inversions between high divergence regions and the genome for each chromosome and the entire, respectively. Chr, chromosome; # inversion, the number of orthologous genes with different orientation between j-Npb and i-9311 genomes; # gene, the total number of orthologous genes between j-Npb and i-9311.
(51 KB DOC)

Table S4 Overrepresentation of Genes Associated with “Biological Process” in High Divergence Regions A total of 398 genes within high divergence genes versus 10,799 genes in rice complete annotation were used in the overrepresentation analysis based on GO “biological process.”
(120 KB DOC)

Table S5 Overrepresentation of Genes Associated with “Molecular Function” in High Divergence Regions A total of 514 genes within high divergence genes versus 14,170 genes in rice complete annotation were used in the overrepresentation analysis based on GO “molecular function.”
(72 KB DOC)

Table S6 Overrepresentation of Genes Associated with “Cellular Component” in High Divergence Regions A total of 202 genes within high divergence genes versus 5,537 genes in rice complete annotation were used in the overrepresentation analysis based on GO “cellular component.”
(71 KB DOC)

Table S7 Protein-Coding Rice Genes That Are Related to Important Phenotype (After Gramene Genes Database, http://www.gramene.org/rice_mutant/index.html) Ks values between j-Npb and i-9311 for each gene were given.
(307 KB DOC)

Table S8 Statistics for Ten Reference Genes in Rice Cultivars and *O. rufipogon,* the Average of Which Is Also Presented in Table 1
π_silent_ is the number of synonymous changes per site averaged over all pair-wise comparisons between sequences, *D_T_* is Tajima's *D* statistic, and *r^2^ ,* the squared allele-frequency correlation, is a common measure of LD.
(63 KB DOC)

### Accession Numbers

The GenBank database (http://www.ncbi.nlm.nih.gov/Genbank) accession numbers for the sequences reported in this paper are *AK064267* (AY886213–AY886237 and DQ374968–DQ374993), *AK068671* (AY886238–AY886269 and DQ374994–DQ375021), *AK069480* (AY886270–AY886299 and DQ375022–DQ375045), *AK069589* (AY886300–AY886331 and DQ375046–DQ375073), *AK071112* (AY886332–AY886362 and DQ375074–DQ375100), *AK100849* (AY886391–AY886421 and DQ375101–DQ375124), *AK100970* (AY886422–AY886447 and DQ375125–DQ375151), *AK102890* (AY886448–AY886477 and DQ375152–DQ375179), *AK105540* (AY886478–AY886508 and DQ375180–DQ375207), *COC1* (AY885916–AY885946 and DQ374829–DQ374856), *EMF1* (AY885887–AY885915 and DQ374801–DQ374828), *GIGANTEA* (AY885710–AY885737 and DQ374670–DQ374697), *HKT1* (AY885801–AY885828 and DQ374748–DQ374774), *MSP1* (AY885857–AY885886 and DQ374775–DQ374800), *Pi-ta* (AY885738–AY885768 and DQ374698–DQ374725), *qSH-1* (AY886122–AY886152 and DQ374913–DQ374940), *RVDE1* (AY886183–AY886212 and DQ374941–DQ374967), *sbe1* (AY886060–AY886090 and DQ374885–DQ374912), *Spl7* (AY885947–AY885975 and DQ374857–DQ374884), and *Xa21* (AY885769–AY885800 and DQ374726–DQ374747).

## Abbreviations

GO: Gene Ontology
i-9311: Oryza sativa ssp. *indica* cv. 9311
j-Npb: Oryza sativa ssp. *japonica* cv. Nipponbare
Kmax: highest Ks value
Ks: number of synonymous substitutions per site
LD: linkage disequilibrium
Mb: megabase
TE: transposable element
