# Supplementary material for: Genomic Variation in Rice: Genesis of Highly Polymorphic Linkage Blocks during Domestication
Source: PLoS Genet. 2006 Nov 17;2(11):e199. doi: 10.1371/journal.pgen.0020199 (PMC1636696; doi:10.1371/journal.pgen.0020199)
Supplement: Supplementary file 1 [file pgen.0020199.sg001.doc]

**Legends of Fig. S1 continue in the next page.**

**Fig. S1.** "Mismatch distributions" of Ks among all 1 Mb segments between j-Npb and i-9311 genomes. A) The observed mismatch distribution; B)-D) simulated distributions under a series of recombination values with 4*Nc*/4*Nμ* =0, 0.0025, and 0.0125. There is no value of 4*Nc* under which the observed and the expected distributions fit. In other words, a population that is not subdivided would not have the observed mismatch distribution under the coalescence framework.

Detailed description: In this figure, we present results from coalescence simulations with recombination. The objective is to see if the number of 1Mb segments that show a divergence > 2.79% is significantly greater than expected in the coalescence framework.

Following convention, we shall refer to the distribution of neutral divergence in Ks among all 1 Mb segments between two genomes as the mismatch distribution. The mismatch distribution depends on the population recombination rate (4*Nc* where *c* is the recombination rate between two adjacent nucleotides and *N* is the effective population size). In Fig. S1a, the observed mismatch distribution between j-Npb and i-9311 is given. Fig. S1b-d present the distribution under a series of recombination values. (The analytical solutions for the mismatch distribution under 4*Nc*=0 and 4*Nc* >> 0 are available but simulations are required for intermediate 4*Nc*’s [see technical details below].)

It is clear that c cannot possibly be 0 in rice, as the observed mode is far removed from the lowest level of divergence required for the geometric distribution under 4*Nc* = 0. When 4*Nc* is very large, the mismatch distribution is Poisson which has all the mass concentrated between 0.010 and 0.015 (not shown; the variance of Poisson is very small for large segments of DNAs) . As 4*Nc* increases, the distribution becomes bimodal with one mode corresponding to that of the geometric distribution and the other (approximately), to that of Poisson. The observed mode is in between the two expected modes. Hence, there is no value of 4*Nc* under which the observed and the expected distributions fit. In other words, a population that is not subdivided would not have the observed mismatch distribution under the coalescence framework.

To get the best fit (albeit still a very poor fit) under the simple coalescence process, we chose the smallest 4*Nc* / 4*Nμ* value (=0.0125) under which the mismatch distribution first becomes unimodal, as 4Nc increases. Fig. S1d shows that, given this recombination rate, the *Ks* value does not exceed 2.2% which is much lower than the cutoff used in Fig. 1 (2.8%). We should note that 4*Nc* / 4*Nμ* = 0.0125 is a very modest value, even for selfing plants. (In nonselfing species such as humans and *Drosophila* for which such ratios have been estimated, the range is 1 – 10.)

In short, demographic models and/or selection hypotheses are needed to account for the presence of high-divergence regions as shown in Fig. 1.

Technical details

1. Formula for the mismatch distribution under 4*Nc* = 0

Let *K* be the number of differences between two sequences. When there is no intra-genic recombination, *K* follows the geometric distribution

$$p\{K=k\}=\left(\frac{\theta}{\theta+1}\right)^{k}\left(\frac{1}{\theta+1}\right),\text{ }k=0,1,2,...$$

where *θ* = 4*Nμ* (*N* is the effective population size, *μ* is the neutral mutation rate of the locus).

2. Simulation protocols

Standard coalescent simulation with recombination *[1,* 2] was used to generated the mismatch distribution when *ρ* (4*Nc*) is not zero (no recombination). In all the simulations, we let *θ* be the mean level of divergence per bp, estimated from the data, but allow *ρ* to vary. For each combination of *θ* and *ρ*, we generated 3587 (the number of windows shown in the sliding window plot) random samples, each of which is 25kb in length. Then we placed these simulated data into bins according to their levels of divergence. Although we have 1Mb per segment, there are on average only 25Kb worth of synonymou sites. However, recombination occurs within the 1 Mb segment. Therefore, the presented *ρ* value is the value used in the simulations divided by 40.

Reference

1.	Hudson RR (1983) Properties of a neutral allele model with intragenic recombination. Theor Popul Biol 23: 183-201.

2.	Hudson RR (2002) Generating samples under a Wright-Fisher neutral model of genetic variation. Bioinformatics 18: 337-338.
