# Supplementary material for: Genomic Variation in Rice: Genesis of Highly Polymorphic Linkage Blocks during Domestication
Source: PLoS Genet. 2006 Nov 17;2(11):e199. doi: 10.1371/journal.pgen.0020199 (PMC1636696; doi:10.1371/journal.pgen.0020199)
Supplement: Supplementary file 2 [file pgen.0020199.st001.doc]

**Table S1.** *Oryza* plant material used in this study.

| Taxa | Code | Accession No. ^a^ | Varieties/Origin |
| --- | --- | --- | --- |
| *O. sativa* spp. *japonica* | R0313 | 30-0041 | Tai Nan #5, Taiwan, China |
| *O. sativa* spp. *japonica* | R0316 | 15-04582* | Ling He, China |
| *O. sativa* spp. *japonica* | R0317 | 15-03898* | Gou Ren Nuo, northern China |
| *O. sativa* spp. *japonica* | R0319 | 15-04015* | 500 Li JiangMi-12, northern China |
| *O. sativa* spp. *japonica* | R0329 | IRGC5745 | DOURADO AGULHA, Brazil |
| *O. sativa* spp. *japonica* | R0336 | IRGC40265 | KUSABUE, Japan |
| *O. sativa* spp. *japonica* | R0337 | IRGC40266 | OUU244, Japan |
| *O. sativa* spp. *japonica* | YueGuang | WD16564 | Yue Guang, Japan |
| *O. sativa* spp. *japonica* | QiuGuang | IRGC76300 | AKIHIKARI, Japan |
| *O. sativa* spp. *japonica* | JinNanFeng | IRGC471 | KIN MAZE, Japan |
| *O. sativa* spp. *japonica* | HuangJinQing | WD29902 | Huang Jin Qing, Japan |
| *O. sativa* spp.*indica* | R0321 | IRGC4895 | BASMATI 370, India |
| *O. sativa* spp.*indica* | R0322 | IRGC5331 | TJAHAJA, Indonesia |
| *O. sativa* spp.*indica* | R0323 | IRGC5323 | BENGAWAN, Indonesia |
| *O. sativa* spp.*indica* | R0324 | IRGC32576 | TETEP, Vietnam |
| *O. sativa* spp.*indica* | R0325 | IRGC5908 | SR 26B, India |
| *O. sativa* spp.*indica* | R0326 | IRGC8944* | ALAGUSAMBA, Sri Lanka |
| *O. sativa* spp.*indica* | R0327 | IRGC7702* | KALUBALA VEE, Sri Lanka |
| *O. sativa* spp.*indica* | R0328 | IRGC9445 | NOVELI 15 IP, Brazil |
| *O. sativa* spp.*indica* | R0332 | IRGC3643* | RAMGARH, India |
| *O. sativa* spp.*indica* | R0333 | IRGC6836 | JKW S 39, Pakistan |
| *O. sativa* spp.*indica* | GLA4 | IRGC59677 | GUANG-LU-AI4, China |
| *O. sativa* spp.*indica* | Os-5 | IRGC8042* | OGNA-PINIDWA, Philippines |
| *O. sativa* spp.*indica* | Os-31 | IRGC67774 | KHAUNG DUM, Myanmar |
| *O. sativa* spp.*indica* | Os-46 | IRGC78236* | BOW KHAO, Thailand |
| *O. rufipogon* | E078 | IRGC105476 | India |
| *O. rufipogon* | RH17 | CNW50117^$^ | Hainan, China |
| *O. rufipogon* | RH28 | CNW50128^$^ | Hainan, China |
| *O. rufipogon* | 2 | CNW50202^$^ | Jiangxi, China |
| *O. rufipogon* | 107 | CNW50307^$^ | Jiangxi, China |
| *O. rufipogon* | 108 | CNW50308^$^ | Jiangxi, China |
| *O. rufipogon* | ORA09 | CNW50409^$^ | Guangdong, China |
| *O. rufipogon* | ORA11 | CNW50411^$^ | Guangdong, China |
| *O. rufipogon* | ORA15 | CNW50415^$^ | Guangdong, China |
| *O. rufipogon* | ORC17 | CNW50417^$^ | Guangdong, China |
| *O. rufipogon* | OR3 | IRGC82040 | Thailand |
| *O. rufipogon* | OR7 | IRGC86542 | Australia |
| *O. rufipogon* | OR11 | IRGC103305 | Philippines |
| *O. rufipogon* | OR12 | IRGC105214 | Sri Lanka |
| *O. rufipogon* | OR13 | IRGC105696 | Nepal |
| *O. rufipogon* | OR15 | IRGC106036 | Malaysia |
| *O. rufipogon* | OR16 | IRGC106146 | Laos |
| *O. rufipogon* | OR17 | IRGC106412 | Vietnam |
| *O. rufipogon* | OR18 | IRGC106453 | Indonesia |
| *O. rufipogon* | OR19 | IRGC106502 | Papua New Guinea |
| *O. barthii* | E3-9 | WYD17 | Mali |
| *O. barthii* | OB125 | IRGC100921 | Africa |
| *O. barthii* | OB136 | IRGC101316 | Guinea |
| *O. meridionalis* | E12-2 | IRGC103319 | Australia |
| *O. meridionalis* | OM301 | IRGC82042 | Australia |
| *O. meridionalis* | OM309 | IRGC105283 | Australia |
| *O. longistaminata* | E11-3 | IRGC101212 | Ivory coast |
| *O. longistaminata* | OL274 | IRGC101205 | Benin |
| *O. longistaminata* | OL285 | IRGC104100 | Botswana |
| *O. glumaepatula* | E8-5 | IRGC100960 | Brazil |
| *O. glumaepatula* | OG196 | IRGC103812 | Venezuela |
| *O. glumaepatula* | OG199 | IRGC105561 | Colombia |
| *O. nivara* | E14-17 | IRGC105417 | Sri Lanka |
| *O. nivara* | ON364 | IRGC103845 | India |
| *O. nivara* | ON365 | IRGC104309 | Thailand |

^a^ *O. sativa* accessions are provided by the Rice Institute of Guangdong and China National Rice Research Institute. Accessions for wild rice outside of China are donated by the International Rice Germplasm Collection (IRGC) at the International Rice Research Institute (IRRI). *O. rufipogon* accessions in China are collected by the authors. Accession numbers follow IRGC codes or the codes of the Institute of Crop Germplasm Resources, Chinese Academy of Agricultural Sciences (CAAS).

* Landrace

^$^ Collected from wild populations.
