# Supplementary material for: Genomic Variation in Rice: Genesis of Highly Polymorphic Linkage Blocks during Domestication
Source: PLoS Genet. 2006 Nov 17;2(11):e199. doi: 10.1371/journal.pgen.0020199 (PMC1636696; doi:10.1371/journal.pgen.0020199)
Supplement: Supplementary file 3 [file pgen.0020199.st002.doc]

**Table S2.** Summary of GC content and TE abundance by sliding window analysis of 1Mb window length and 0.1Mb intervals across Nipponbare genome.

|  | Comparison between regions | | Correlation with Ks ^b^  (*r^2^*) |
| --- | --- | --- | --- |
|  | High divergence regions | Rest of the genome |  |
| GC Content | 0.44±0.1 | 0.43±0.1 | 0.013** |
| TE abundance ^a^ | 0.04±0.11 **  (47.23±42.49 ***) | 0.04±0.09  (48.15±15.37) | 0.007**  (0.017 **) |

^a^ TE abundance was estimated by measuring the fraction of DNA occupied by TEs (upper values) and by counting the number of TEs ( values in parentheses) in each 1-Mb sliding window. Significance levels are shown for comparisons between the high divergence regions and the rest of the genome by the Mann-Whitney U test.

^b^ All Pearson’s correlation coefficient (*r*) are significant at the 0.01 level.

Two-tailed significance:*, P <0.05; **, P <0.01; ***, P <0.001.
