# Supplementary material for: Genomic Variation in Rice: Genesis of Highly Polymorphic Linkage Blocks during Domestication
Source: PLoS Genet. 2006 Nov 17;2(11):e199. doi: 10.1371/journal.pgen.0020199 (PMC1636696; doi:10.1371/journal.pgen.0020199)
Supplement: Supplementary file 4 [file pgen.0020199.st003.doc]

**Table S3.** The abundance of inversions between j-Npb and i-9311 in high divergence regions and the whole genome. High divergence regions are distributed on chromosome 4, 6, 7, 10, 11, and 12. Fisher’s exact tests were conducted to compare the abundance of inversions between high divergence regions and the genome for each chromosome and the entire, respectively. Chr., chromosome; # inversion, the number of orthologous genes with different orientation between j-Npb and i-9311 genomes; # gene, the total number of orthologous genes between j-Npb and i-9311.

| Chr. | High divergence region | | Genome | |
| --- | --- | --- | --- | --- |
|  | # inversion ^a^ | # gene | # inversion | # gene |
| 1 | 0 | 0 | 29 | 4237 |
| j2 | 0 | 0 | 45 | 3331 |
| 3 | 0 | 0 | 39 | 3774 |
| 4 | 23 | 314 | 146 | 2731 |
| 5 | 0 | 0 | 36 | 2358 |
| 6 | 2 | 67 | 35 | 2559 |
| 7 | 3 | 78 | 38 | 2428 |
| 8 | 0 | 0 | 26 | 2190 |
| 9 | 0 | 0 | 11 | 1718 |
| 10 | 0 | 174 | 17 | 1812 |
| 11 | 81*** | 274 | 134 | 2108 |
| 12 | 8 | 252 | 43 | 1777 |
| Total | 117*** | 1159 | 599 | 31023 |

^a^ As we aim to assess the correlation between inversion and diversity, we only focus on genes that appear to have complete inversion between j-Npb and i-9311, i.e. orthologous genes with different orientation between j-Npb and i-9311 genomes. Inversions contained within gene or overlapped with gene were not taken into consideration, because it’s hard to say whether inversions occurring in different position or/and portion of gene have the same effects on diversity.

Fisher’s exact tests: *, *p*<0.05; **, *p*<0.01; ***, *p*<0.001.
