# Supplementary material for: Genomic Variation in Rice: Genesis of Highly Polymorphic Linkage Blocks during Domestication
Source: PLoS Genet. 2006 Nov 17;2(11):e199. doi: 10.1371/journal.pgen.0020199 (PMC1636696; doi:10.1371/journal.pgen.0020199)
Supplement: Supplementary file 5 [file pgen.0020199.st004.doc]

**Table S4.** Overrepresentation of genes associated with “biological process” in high divergence regions. A total of 398 genes within high divergence genes versus 10,799 genes in rice complete annotation were used in the over-representation analysis based on gene ontology “biological process”.

| **Biological process ^a^**  **(n=398)** | **Observed** | **Expected ^b^** | ***p*-value ^c^** | **Corrected *p*-value ^d^** |
| --- | --- | --- | --- | --- |
| Response to biotic stimulus | 190 | 94.35 | **** | **** |
| Biopolymer modification | 128 | 72.42 | **** | **** |
| Protein modification | 128 | 72.42 | **** | **** |
| Signal transduction | 127 | 74.41 | **** | **** |
| Response to stimulus | 225 | 162.90 | **** | **** |
| Biopolymer metabolism | 131 | 80.71 | **** | **** |
| Response to endogenous stimulus | 122 | 73.71 | **** | **** |
| Cell communication | 129 | 80.27 | **** | **** |
| Cellular protein metabolism | 140 | 90.26 | **** | **** |
| Cellular macromolecule metabolism | 140 | 90.26 | **** | **** |
| Protein metabolism | 163 | 126.30 | **** | *** |
| Cell differentiation | 45 | 26.65 | *** | ** |
| Macromolecule metabolism | 190 | 160.21 | ** | ** |
| Flower development | 24 | 13.89 | ** | * |
| Pollination | 5 | 2.87 | NS | NS |
| Reproductive organismal physiological process | 5 | 2.87 | NS | NS |
| Reproductive physiological process | 5 | 2.87 | NS | NS |
| Cell death | 14 | 10.47 | NS | NS |
| Post-embryonic development | 24 | 19.64 | NS | NS |
| Organismal physiological process | 5 | 3.02 | NS | NS |
| Behavior | 15 | 11.90 | NS | NS |
| Cellular metabolism | 197 | 194.60 | NS | NS |
| Pollen-pistil interaction | 1 | 0.70 | NS | NS |
| Morphogenesis | 23 | 22.96 | NS | NS |
| Lipid metabolism | 41 | 41.76 | NS | NS |
| Secondary metabolism | 34 | 34.75 | NS | NS |
| Growth | 26 | 26.76 | NS | NS |
| Embryonic development | 1 | 0.96 | NS | NS |
| Primary metabolism | 242 | 245.20 | NS | NS |
| Carbohydrate metabolism | 24 | 26.06 | NS | NS |
| Development | 55 | 58.16 | NS | NS |
| Metabolism | 275 | 280.25 | NS | NS |
| Cell cycle | 6 | 7.44 | NS | NS |
| Response to external stimulus | 46 | 50.23 | NS | NS |
| Ripening | 1 | 1.70 | NS | NS |
| Reproductive structure development | 1 | 1.70 | NS | NS |
| Reproduction | 8 | 10.47 | NS | NS |
| Death | 17 | 20.79 | NS | NS |
| Cellular physiological process | 249 | 260.12 | NS | NS |
| Amino acid and derivative metabolism | 32 | 40.58 | NS | NS |
| Lipid binding | 5 | 8.77 | NS | NS |
| Obsolete biological process | 5 | 8.77 | NS | NS |
| Cellular process | 253 | 269.52 | NS | NS |
| Protein biosynthesis | 12 | 19.09 | NS | NS |
| Macromolecule biosynthesis | 12 | 19.13 | NS | NS |
| Cellular biosynthesis | 12 | 19.13 | NS | NS |
| Biosynthesis | 66 | 82.85 | NS | NS |
| DNA metabolism | 3 | 8.96 | NS | NS |
| Response to extracellular stimulus | 1 | 5.09 | NS | NS |
| Response to abiotic stimulus | 36 | 53.96 | NS | NS |
| Response to stress | 55 | 76.95 | NS | NS |
| Electron transport | 9 | 22.85 | NS | NS |
| Cell organization and biogenesis | 8 | 22.26 | NS | NS |
| Physiological process | 316 | 342.16 | NS | NS |
| Transport | 40 | 65.97 | NS | NS |
| Localization | 40 | 65.97 | NS | NS |
| Establishment of localization | 40 | 65.97 | NS | NS |
| Generation of precursor metabolites and energy | 10 | 26.57 | NS | NS |
| Catabolism | 17 | 38.26 | NS | NS |
| Transcription | 15 | 35.57 | NS | NS |
| Nucleobase, nucleoside, nucleotide and nucleic acid metabolism | 27 | 56.09 | NS | NS |

^a^ Note that one gene might be involved in multiple biological process. A total of 398 annotated genes associated with “biological process” within high divergence genes were analyzed.

^b^ The expected number of genes within high divergence regions for a given functional category was computed by 398m/10799, where m is the number of annotated genes belonging to the functional category, 10,799 is the total number of annotated rice genes and 398 is the total number of annotated genes within high divergence regions.

^c^ Determined by Hypergeometric test.

^d^ Benjamini & Hochberg False Discovery Rate (FDR) correction.

NS, not significant; *, overrepresented with *p*< 0.05; **, overrepresented with *p*< 0.01; *** overrepresented with *p*< 0.001; **** overrepresented with *p*< 0.0001.
