# Supplementary material for: Genomic Variation in Rice: Genesis of Highly Polymorphic Linkage Blocks during Domestication
Source: PLoS Genet. 2006 Nov 17;2(11):e199. doi: 10.1371/journal.pgen.0020199 (PMC1636696; doi:10.1371/journal.pgen.0020199)
Supplement: Supplementary file 6 [file pgen.0020199.st005.doc]

**Table S5.** Overrepresentation of genes associated with “molecular function” in high divergence regions. A total of 514 genes within high divergence genes versus 14,170 genes in rice complete annotation were used in the over-representation analysis based on gene ontology “molecular function”.

| **Molecular function ^a^**  **(n=514)** | **Observed** | **Expected ^b^** | ***p*-value ^c^** | **Corrected *p*-value ^d^** |
| --- | --- | --- | --- | --- |
| Carbohydrate binding | 104 | 40.08 | **** | **** |
| Receptor activity | 47 | 14.26 | **** | **** |
| Kinase activity | 146 | 84.34 | **** | **** |
| Transferase activity, transferring phosphorus-containing groups | 146 | 84.41 | **** | **** |
| Nucleotide binding | 145 | 88.36 | **** | **** |
| Protein binding | 157 | 100.77 | **** | **** |
| Signal transducer activity | 61 | 27.57 | **** | **** |
| Transferase activity | 187 | 137.08 | **** | **** |
| Catalytic activity | 329 | 303.61 | * | * |
| Nuclease activity | 9 | 4.50 | * | NS |
| Hydrolase activity, acting on ester bonds | 9 | 4.57 | * | NS |
| Binding | 284 | 266.98 | NS | NS |
| Hydrolase activity | 114 | 106.65 | NS | NS |
| RNA binding | 17 | 15.71 | NS | NS |
| Motor activity | 4 | 3.52 | NS | NS |
| Enzyme regulator activity | 7 | 6.93 | NS | NS |
| Obsolete molecular function | 5 | 6.49 | NS | NS |
| Chaperone activity | 5 | 6.49 | NS | NS |
| Translation regulator activity | 2 | 4.14 | NS | NS |
| Translation factor activity, nucleic acid binding | 2 | 4.14 | NS | NS |
| Lipid binding | 5 | 8.63 | NS | NS |
| Oxygen binding | 8 | 14.11 | NS | NS |
| Structural molecule activity | 6 | 12.70 | NS | NS |
| Transcription factor activity | 22 | 38.34 | NS | NS |
| Transcription regulator activity | 22 | 43.24 | NS | NS |
| Transporter activity | 24 | 48.21 | NS | NS |
| DNA binding | 38 | 67.58 | NS | NS |
| Nucleic acid binding | 61 | 97.50 | NS | NS |

^a^ Note that one gene might have multiple molecular functions. A total of 514 annotated genes associated with “molecular function” within high divergence genes were analyzed.

^b^ The expected number of genes within high divergence regions for a given functional category was computed by 514m/14170, where m is the number of annotated genes belonging to the functional category, 14,170 is the total number of annotated rice genes and 514 is the total number of annotated genes within high divergence regions.

^c^ Determined by Hypergeometric test.

^d^ Benjamini & Hochberg False Discovery Rate (FDR) correction.

NS, not significant; *, overrepresented with *p*< 0.05; **, overrepresented with *p*< 0.01; *** overrepresented with *p*< 0.001; **** overrepresented with *p*< 0.0001.
