# Supplementary material for: Genomic Variation in Rice: Genesis of Highly Polymorphic Linkage Blocks during Domestication
Source: PLoS Genet. 2006 Nov 17;2(11):e199. doi: 10.1371/journal.pgen.0020199 (PMC1636696; doi:10.1371/journal.pgen.0020199)
Supplement: Supplementary file 7 [file pgen.0020199.st006.doc]

**Table S6.** Overrepresentation of genes associated with “cellular component” in high divergence regions. A total of 202 genes within high divergence genes versus 5,537 genes in rice complete annotation were used in the over-representation analysis based on gene ontology “cellular component”.

| **Cellular component ^a^**  **(n=202)** | **Observed** | **Expected ^b^** | ***p*-value ^c^** | **Corrected *p*-value ^d^** |
| --- | --- | --- | --- | --- |
| Cell wall | 63 | 18.24 | **** | **** |
| External encapsulating structure | 63 | 18.24 | **** | **** |
| Plasma membrane | 91 | 40.42 | **** | **** |
| Membrane | 142 | 109.85 | **** | **** |
| Cell part | 195 | 189.34 | NS | NS |
| Cell | 196 | 191.06 | NS | NS |
| Cytosol | 15 | 10.43 | NS | NS |
| Microbody | 2 | 2.04 | NS | NS |
| Peroxisome | 2 | 2.04 | NS | NS |
| Extracellular region | 7 | 9.16 | NS | NS |
| Endoplasmic reticulum | 13 | 17.66 | NS | NS |
| Mitochondrion | 6 | 9.52 | NS | NS |
| Plastid | 15 | 21.41 | NS | NS |
| Ribosome | 6 | 10.54 | NS | NS |
| Ribonucleoprotein complex | 6 | 10.54 | NS | NS |
| Protein complex | 6 | 13.97 | NS | NS |
| Intracellular non-membrane-bound organelle | 6 | 14.19 | NS | NS |
| Non-membrane-bound organelle | 6 | 14.19 | NS | NS |
| Cytoplasmic part | 42 | 58.37 | NS | NS |
| Cytoplasm | 46 | 63.26 | NS | NS |
| Thylakoid | 2 | 8.03 | NS | NS |
| Nucleus | 13 | 29.73 | NS | NS |
| Intracellular membrane-bound organelle | 39 | 71.36 | NS | NS |
| Membrane-bound organelle | 39 | 71.36 | NS | NS |
| Intracellular part | 54 | 90.18 | NS | NS |
| Organelle | 45 | 83.43 | NS | NS |
| Intracellular organelle | 45 | 83.43 | NS | NS |

^a^ Note that one gene might have multiple molecular functions. A total of 202 annotated genes associated with “molecular function” within high divergence genes were analyzed.

^b^ The expected number of genes within high divergence regions for a given functional category was computed by 202m/5537, where m is the number of annotated genes belonging to the functional category, 5537 is the total number of annotated rice genes and 202 is the total number of annotated genes within high divergence regions.

^c^ Determined by Hypergeometric test.

^d^ Benjamini & Hochberg False Discovery Rate (FDR) correction.

NS, not significant; *, overrepresented with *p*< 0.05; **, overrepresented with *p*< 0.01; *** overrepresented with *p*< 0.001; **** overrepresented with *p*< 0.0001.
