# Supplementary material for: Genomic Variation in Rice: Genesis of Highly Polymorphic Linkage Blocks during Domestication
Source: PLoS Genet. 2006 Nov 17;2(11):e199. doi: 10.1371/journal.pgen.0020199 (PMC1636696; doi:10.1371/journal.pgen.0020199)
Supplement: Supplementary file 8 [file pgen.0020199.st007.doc]

**Table S7. Protien-coding rice genes that are related to important phenotype (after Gramene Genes Database,** [**http://www.gramene.org/rice_mutant/index.html**](http://www.gramene.org/rice_mutant/index.html)**). Ks value between j-Npb and i-9311 for each gene were given.**

| [**Gene Symbol**](http://www.gramene.org/db/genes/search_gene?query=*&search_field=name&species=1&gene_type_id=2&has_phenotype=&query_submit=Search&order_by=symbol) | **Gene Ontology** | [**Chr. No.**](http://www.gramene.org/db/genes/search_gene?query=*&search_field=name&species=1&gene_type_id=2&has_phenotype=&query_submit=Search&order_by=chromosome) | **Gramene Accession** | **Rice Ensembl Gene** | **Ks** |
| --- | --- | --- | --- | --- | --- |
| [*ACC1*](http://www.gramene.org/db/genes/search_gene?acc=GR:0060004) | 1-aminocyclopropane-1-carboxylate synthase activity, transaminase activity, biosynthesis, cytoplasm | 3 | GR:0060004 | [LOC_Os03g51740](http://www.gramene.org/Oryza_sativa/geneview?gene=LOC_Os03g51740) | 0 |
| [*Amy4*](http://www.gramene.org/db/genes/search_gene?acc=GR:0060045) | cytoplasmic membrane-bound vesicle, sucrose catabolism, cellular component unknown, secretory pathway, starch catabolism, alpha-amylase activity | 9 | GR:0060045 | [LOC_Os06g49970](http://www.tigr.org/tigr-scripts/euk_manatee/BacAnnotationPage.cgi?db=osa1&asmbl_id=11976) | 0.00734 |
| [*atpB1*](http://www.gramene.org/db/genes/search_gene?acc=GR:0100189) | hydrogen-transporting ATP synthase activity, rotational mechanism, proton-transporting ATP synthase, catalytic core (sensu Eukaryota), mitochondrion, proton-transporting two-sector ATPase complex, ATP synthesis coupled proton transport, ATP biosynthesis, ATP binding | 5 | GR:0100189 | [LOC_Os05g47980](http://www.gramene.org/Oryza_sativa/geneview?gene=LOC_Os05g47980) | 0.01602 |
| [*atpG*](http://www.gramene.org/db/genes/search_gene?acc=GR:0100191) | hydrogen-transporting ATP synthase activity, rotational mechanism, proton-transporting ATP synthase, catalytic core (sensu Eukaryota), hydrogen-transporting ATPase activity, rotational mechanism, proton-transporting two-sector ATPase complex, membrane, hydrogen transport, ATP synthesis coupled proton transport, ATP biosynthesis | 10 | GR:0100191 | [LOC_Os10g17280](http://www.gramene.org/Oryza_sativa/geneview?gene=LOC_Os10g17280) | 0 |
| [*bc1*](http://www.gramene.org/db/genes/search_gene?acc=GR:0060068) | secondary cell wall biosynthesis (sensu Magnoliophyta), cell wall modification during abscission, lignin metabolism, secondary cell wall | 3 | GR:0060068 | [LOC_Os03g30250](http://www.gramene.org/Oryza_sativa/geneview?gene=LOC_Os03g30250) | 0.00289 |
| [*bierf1*](http://www.gramene.org/db/genes/search_gene?acc=GR:0020001) | response to pathogenic fungi, cytoplasmic membrane-bound vesicle, mitochondrion, transcription factor activity, nucleus, plastid, regulation of transcription, DNA-dependent, secretory pathway | 9 | GR:0020001 | [LOC_Os09g26420](http://www.gramene.org/Oryza_sativa/geneview?gene=LOC_Os09g26420) | 0.01161 |
| [*bierf2*](http://www.gramene.org/db/genes/search_gene?acc=GR:0020002) | transcription factor activity, nucleus, plastid, regulation of transcription, DNA-dependent | 1 | GR:0020002 | [LOC_Os01g58420](http://www.gramene.org/Oryza_sativa/geneview?gene=LOC_Os01g58420) | 0.01325 |
| [*bierf3*](http://www.gramene.org/db/genes/search_gene?acc=GR:0020003) | response to pathogenic fungi, transcription factor activity, nucleus, regulation of transcription, DNA-dependent, secretory pathway | 2 | GR:0020003 | [LOC_Os02g43790](http://www.gramene.org/Oryza_sativa/geneview?gene=LOC_Os02g43790) | 0 |
| [*bierf4*](http://www.gramene.org/db/genes/search_gene?acc=GR:0020004) | transcription factor activity, nucleus, plastid, regulation of transcription, DNA-dependent | 3 | GR:0020004 | [LOC_Os03g08500](http://www.gramene.org/Oryza_sativa/geneview?gene=LOC_Os03g08500) | 0 |
| [*ckx2*](http://www.gramene.org/db/genes/search_gene?acc=GR:0020051) | cytoplasmic membrane-bound vesicle, inflorescence development, cytokinin dehydrogenase activity, electron transport, secretory pathway | 1 | GR:0020051 | [LOC_Os01g10110](http://www.gramene.org/Oryza_sativa/geneview?gene=LOC_Os01g10110) | 0 |
| [*ckx3*](http://www.gramene.org/db/genes/search_gene?acc=GR:0020052) | cytoplasmic membrane-bound vesicle, cytokinin dehydrogenase activity, oxidoreductase activity, electron transport, secretory pathway | 10 | GR:0020052 | [LOC_Os10g34230](http://www.gramene.org/Oryza_sativa/geneview?gene=LOC_Os10g34230) | 0 |
| [*ckx4*](http://www.gramene.org/db/genes/search_gene?acc=GR:0020053) | cytoplasmic membrane-bound vesicle, cytokinin dehydrogenase activity, oxidoreductase activity, electron transport, secretory pathway | 1 | GR:0020053 | [LOC_Os01g71310](http://www.gramene.org/Oryza_sativa/geneview?gene=LOC_Os01g71310) | 0.00179 |
| [*ckx5*](http://www.gramene.org/db/genes/search_gene?acc=GR:0020054) | cytoplasmic membrane-bound vesicle, cytokinin dehydrogenase activity, oxidoreductase activity, electron transport, plastid, secretory pathway | 1 | GR:0020054 | [LOC_Os01g56810](http://www.gramene.org/Oryza_sativa/geneview?gene=LOC_Os01g56810) | 0.00181 |
| [*cox15*](http://www.gramene.org/db/genes/search_gene?acc=GR:0100135) | mitochondrial electron transport, cytochrome c to oxygen, respiratory chain complex IV (sensu Eukaryota), mitochondrion, cytochrome-c oxidase activity, protein complex assembly, membrane, aerobic respiration | 8 | GR:0100135 | [LOC_Os08g38720](http://www.gramene.org/Oryza_sativa/geneview?gene=LOC_Os08g38720) | 0 |
| [*cox5b*](http://www.gramene.org/db/genes/search_gene?acc=GR:0100133) | mitochondrial electron transport, cytochrome c to oxygen, mitochondrial envelope, respiratory chain complex IV (sensu Eukaryota), mitochondrion, electron transport, cytochrome-c oxidase activity, aerobic respiration | 1 | GR:0100133 | [LOC_Os01g42650](http://www.gramene.org/Oryza_sativa/geneview?gene=LOC_Os01g42650) | 0.00876 |
| [*cox5c*](http://www.gramene.org/db/genes/search_gene?acc=GR:0100134) | mitochondrial electron transport, cytochrome c to oxygen, respiratory chain complex IV (sensu Eukaryota), mitochondrion, cytochrome-c oxidase activity, aerobic respiration | 12 | GR:0100134 | [LOC_Os12g37420](http://www.gramene.org/Oryza_sativa/geneview?gene=LOC_Os12g37420) | 0 |
| [*cox6b*](http://www.gramene.org/db/genes/search_gene?acc=GR:0061299) | mitochondrial electron transport, cytochrome c to oxygen, respiratory chain complex IV (sensu Eukaryota), mitochondrion, electron transport, cytochrome-c oxidase activity, aerobic respiration | 3 | GR:0061299 | [LOC_Os03g27290](http://www.gramene.org/Oryza_sativa/geneview?gene=LOC_Os03g27290) | 0.01375 |
| [*cps1*](http://www.gramene.org/db/genes/search_gene?acc=GR:0020064) | lyase activity, metabolism, gibberellic acid metabolism, plastid | 2 | GR:0020064 | [LOC_Os02g17780](http://www.gramene.org/Oryza_sativa/geneview?gene=LOC_Os02g17780) | 0.00434 |
| [*cpsl3*](http://www.gramene.org/db/genes/search_gene?acc=GR:0020066) | lyase activity, metabolism, gibberellic acid metabolism, plastid | 4 | GR:0020066 | [LOC_Os04g09900](http://www.gramene.org/Oryza_sativa/geneview?gene=LOC_Os04g09900) | 0.02934 |
| [*cytc1*](http://www.gramene.org/db/genes/search_gene?acc=GR:0100186) | electron carrier activity, mitochondrion, electron transport, respiratory chain complex III (sensu Eukaryota), plastid, mitochondrial electron transport, ubiquinol to cytochrome c, mitochondrial electron transport chain, electron transporter, transferring electrons within CoQH2-cytochrome c reductase complex activity | 1 | GR:0100186 | [LOC_Os01g70960](http://www.gramene.org/Oryza_sativa/geneview?gene=LOC_Os01g70960) | 0 |
| [*d1*](http://www.gramene.org/db/genes/search_gene?acc=GR:0060184) | G-protein coupled receptor protein signaling pathway, defense response to pathogen, GTPase activity, heterotrimeric G-protein complex, signal transducer activity, GTP binding, development, gibberellic acid mediated signaling, G-alpha-dependent | 5 | GR:0060184 | [LOC_Os05g26890](http://www.gramene.org/Oryza_sativa/geneview?gene=LOC_Os05g26890) | 0.00361 |
| [*d2*](http://www.gramene.org/db/genes/search_gene?acc=GR:0060185) | cytoplasmic membrane-bound vesicle, mitochondrion, electron transport, development, unidimensional cell growth, secretory pathway | 1 | GR:0060185 | [LOC_Os03g40540](http://www.tigr.org/tigr-scripts/euk_manatee/BacAnnotationPage.cgi?db=osa1&asmbl_id=11973) | 0.01226 |
| [*d35*](http://www.gramene.org/db/genes/search_gene?acc=GR:0060213) | cytoplasmic membrane-bound vesicle, ent-kaurene oxidase activity, gibberellic acid biosynthesis, electron transport, secretory pathway | 6 | GR:0060213 | [LOC_Os06g37300](http://www.gramene.org/Oryza_sativa/geneview?gene=LOC_Os06g37300) | 0 |
| [*dl1*](http://www.gramene.org/db/genes/search_gene?acc=GR:0060233) | cell differentiation, meristem determinacy, leaf development, carpel development | 3 | GR:0060233 | [LOC_Os03g11600](http://www.gramene.org/Oryza_sativa/geneview?gene=LOC_Os03g11600) | 0.00455 |
| [*Em*](http://www.gramene.org/db/genes/search_gene?acc=GR:0060291) | response to abscisic acid stimulus | 5 | GR:0060291 | [LOC_Os05g28210](http://www.gramene.org/Oryza_sativa/geneview?gene=LOC_Os05g28210) | 0.015 |
| [*Fl1*](http://www.gramene.org/db/genes/search_gene?acc=GR:0060860) | biological process unknown, DNA binding, long-day photoperiodism, inflorescence development, transcription factor activity, nucleus, short-day photoperiodism, zinc ion binding, photoperiodism | 6 | GR:0060860 | [LOC_Os06g16370](http://www.gramene.org/Oryza_sativa/geneview?gene=LOC_Os06g16370) | 0 |
| [*Fl32a*](http://www.gramene.org/db/genes/search_gene?acc=GR:0060442) | phosphatidylethanolamine binding, inflorescence development, biological process unknown, transcription factor activity, short-day photoperiodism, regulation of timing of transition from vegetative to reproductive phase, photoperiodism | 6 | GR:0060442 | [LOC_Os06g06320](http://www.gramene.org/Oryza_sativa/geneview?gene=LOC_Os06g06320) | 0.00787 |
| [*Fl34*](http://www.gramene.org/db/genes/search_gene?acc=GR:0060444) | biological process unknown, protein-tyrosine kinase activity, protein kinase activity, ATP binding, inflorescence development, protein amino acid phosphorylation, transcription factor activity, protein kinase CK2 activity, photoperiodism | 3 | GR:0060444 | [LOC_Os03g55389](http://www.tigr.org/tigr-scripts/euk_manatee/BacAnnotationPage.cgi?db=osa1&asmbl_id=11973) | 0 |
| [*Fl8*](http://www.gramene.org/db/genes/search_gene?acc=GR:0060864) | heme oxygenase (decyclizing) activity, inflorescence development, biological process unknown, heme oxidation, transcription factor activity, plastid, photoperiodism | 6 | GR:0060864 | [LOC_Os06g40080](http://www.gramene.org/Oryza_sativa/geneview?gene=LOC_Os06g40080) | 0 |
| [*ga20ox1*](http://www.gramene.org/db/genes/search_gene?acc=GR:0020075) | gibberellic acid metabolism | 3 | GR:0020075 | [LOC_Os03g63970](http://www.gramene.org/Oryza_sativa/geneview?gene=LOC_Os03g63970) | 0.00461 |
| [*ga2ox-1*](http://www.gramene.org/db/genes/search_gene?acc=GR:0020077) | antibiotic biosynthesis, isopenicillin-N synthase activity, gibberellic acid metabolism, iron ion binding | 5 | GR:0020077 | [LOC_Os05g06670](http://www.gramene.org/Oryza_sativa/geneview?gene=LOC_Os05g06670) | 0 |
| [*ga3ox2*](http://www.gramene.org/db/genes/search_gene?acc=GR:0020076) | gibberellic acid metabolism | 1 | GR:0020076 | [LOC_Os01g08220](http://www.gramene.org/Oryza_sativa/geneview?gene=LOC_Os01g08220) | 0.01641 |
| [*gh2*](http://www.gramene.org/db/genes/search_gene?acc=GR:0060366) | electron carrier activity, electron transport, lignin biosynthesis, cinnamyl-alcohol dehydrogenase activity, heme binding, zinc ion binding, alcohol dehydrogenase activity, zinc-dependent | 2 | GR:0060366 | [LOC_Os02g09490](http://www.gramene.org/Oryza_sativa/geneview?gene=LOC_Os02g09490) | 0.00395 |
| [*gh3.1*](http://www.gramene.org/db/genes/search_gene?acc=GR:0020037) | response to auxin stimulus, response to light stimulus | 1 | GR:0020037 | [LOC_Os01g57610](http://www.gramene.org/Oryza_sativa/geneview?gene=LOC_Os01g57610) | 0.00181 |
| [*gh3.10*](http://www.gramene.org/db/genes/search_gene?acc=GR:0020046) | response to auxin stimulus, response to light stimulus, plastid | 7 | GR:0020046 | [LOC_Os07g38860](http://www.gramene.org/Oryza_sativa/geneview?gene=LOC_Os07g38860) | 0.00246 |
| [*gh3.11*](http://www.gramene.org/db/genes/search_gene?acc=GR:0020047) | response to auxin stimulus, response to light stimulus | 7 | GR:0020047 | [LOC_Os07g47490](http://www.gramene.org/Oryza_sativa/geneview?gene=LOC_Os07g47490) | 0.00192 |
| [*gh3.12*](http://www.gramene.org/db/genes/search_gene?acc=GR:0020048) | response to auxin stimulus, response to light stimulus | 11 | GR:0020048 | [LOC_Os11g08340](http://www.gramene.org/Oryza_sativa/geneview?gene=LOC_Os11g08340) | 0.00895 |
| [*gh3.13*](http://www.gramene.org/db/genes/search_gene?acc=GR:0020049) | response to auxin stimulus, response to light stimulus, plastid | 11 | GR:0020049 | [LOC_Os11g32520](http://www.gramene.org/Oryza_sativa/geneview?gene=LOC_Os11g32520) | 0.01458 |
| [*gh3.2*](http://www.gramene.org/db/genes/search_gene?acc=GR:0020038) | cytoplasmic membrane-bound vesicle, response to auxin stimulus, response to light stimulus, secretory pathway | 1 | GR:0020038 | [LOC_Os01g55940](http://www.gramene.org/Oryza_sativa/geneview?gene=LOC_Os01g55940) | 0.00194 |
| [*gh3.3*](http://www.gramene.org/db/genes/search_gene?acc=GR:0020039) | response to auxin stimulus, response to light stimulus | 1 | GR:0020039 | [LOC_Os01g12160](http://www.ncbi.nlm.nih.gov/entrez/query.fcgi?cmd=search&db=nucleotide&term=LOC_Os01g12160%5Baccn%5D) | 0 |
| [*gh3.4*](http://www.gramene.org/db/genes/search_gene?acc=GR:0020040) | response to auxin stimulus, response to light stimulus, plastid | 5 | GR:0020040 | [LOC_Os05g42150](http://www.gramene.org/Oryza_sativa/geneview?gene=LOC_Os05g42150) | 0 |
| [*gh3.5*](http://www.gramene.org/db/genes/search_gene?acc=GR:0020041) | response to auxin stimulus, response to light stimulus | 5 | GR:0020041 | [LOC_Os05g50890](http://www.gramene.org/Oryza_sativa/geneview?gene=LOC_Os05g50890) | 0.00733 |
| [*gh3.6*](http://www.gramene.org/db/genes/search_gene?acc=GR:0020042) | response to auxin stimulus, mitochondrion, response to light stimulus, plastid | 5 | GR:0020042 | [LOC_Os05g05180](http://www.gramene.org/Oryza_sativa/geneview?gene=LOC_Os05g05180) | 0.00117 |
| [*gh3.7*](http://www.gramene.org/db/genes/search_gene?acc=GR:0020043) | response to auxin stimulus, response to light stimulus | 6 | GR:0020043 | [LOC_Os06g30440](http://www.gramene.org/Oryza_sativa/geneview?gene=LOC_Os06g30440) | 0.00454 |
| [*gh3.8*](http://www.gramene.org/db/genes/search_gene?acc=GR:0020044) | response to auxin stimulus, response to light stimulus | 7 | GR:0020044 | [LOC_Os07g40290](http://www.gramene.org/Oryza_sativa/geneview?gene=LOC_Os07g40290) | 0 |
| [*gh3.9*](http://www.gramene.org/db/genes/search_gene?acc=GR:0020045) | response to auxin stimulus, response to light stimulus | 7 | GR:0020045 | [LOC_Os07g38890](http://www.gramene.org/Oryza_sativa/geneview?gene=LOC_Os07g38890) | 0.00594 |
| [*Gi*](http://www.gramene.org/db/genes/search_gene?acc=GR:0100114) | inflorescence development, nucleus | 1 | GR:0100114 | [LOC_Os01g08700](http://www.gramene.org/Oryza_sativa/geneview?gene=LOC_Os01g08700) | 0.00325 |
| [*gid1*](http://www.gramene.org/db/genes/search_gene?acc=GR:0020050) | response to gibberellic acid stimulus, nucleus | 5 | GR:0020050 | [LOC_Os05g33730](http://www.gramene.org/Oryza_sativa/geneview?gene=LOC_Os05g33730) | 0.00933 |
| [*iaa1*](http://www.gramene.org/db/genes/search_gene?acc=GR:0020005) | response to auxin stimulus, protein dimerization activity, nucleus, regulation of transcription, regulation of translation | 1 | GR:0020005 | [LOC_Os01g08320](http://www.gramene.org/Oryza_sativa/geneview?gene=LOC_Os01g08320) | 0 |
| [*iaa10*](http://www.gramene.org/db/genes/search_gene?acc=GR:0020014) | response to auxin stimulus, protein dimerization activity, nucleus, regulation of transcription, regulation of translation | 2 | GR:0020014 | [LOC_Os02g57250](http://www.gramene.org/Oryza_sativa/geneview?gene=LOC_Os02g57250) | 0 |
| [*iaa11*](http://www.gramene.org/db/genes/search_gene?acc=GR:0020015) | response to auxin stimulus, protein dimerization activity, nucleus, regulation of transcription, regulation of translation | 3 | GR:0020015 | [LOC_Os03g43400](http://www.gramene.org/Oryza_sativa/geneview?gene=LOC_Os03g43400) | 0 |
| [*iaa12*](http://www.gramene.org/db/genes/search_gene?acc=GR:0020017) | response to auxin stimulus, protein dimerization activity, nucleus, regulation of transcription, regulation of translation | 3 | GR:0020017 | [LOC_Os03g43410](http://www.gramene.org/Oryza_sativa/geneview?gene=LOC_Os03g43410) | 0 |
| [*iaa13*](http://www.gramene.org/db/genes/search_gene?acc=GR:0020016) | response to auxin stimulus, response to light stimulus, protein dimerization activity, nucleus, regulation of transcription, regulation of translation | 3 | GR:0020016 | [LOC_Os03g53150](http://www.gramene.org/Oryza_sativa/geneview?gene=LOC_Os03g53150) | 0 |
| [*iaa14*](http://www.gramene.org/db/genes/search_gene?acc=GR:0020018) | response to auxin stimulus, protein dimerization activity, nucleus, regulation of transcription, regulation of translation | 3 | GR:0020018 | [LOC_Os03g58350](http://www.gramene.org/Oryza_sativa/geneview?gene=LOC_Os03g58350) | 0.00916 |
| [*iaa15*](http://www.gramene.org/db/genes/search_gene?acc=GR:0020019) | response to auxin stimulus, protein dimerization activity, nucleus, plastid, regulation of transcription, regulation of translation | 5 | GR:0020019 | [LOC_Os05g08570](http://www.gramene.org/Oryza_sativa/geneview?gene=LOC_Os05g08570) | 0 |
| [*iaa16*](http://www.gramene.org/db/genes/search_gene?acc=GR:0020020) | response to auxin stimulus | 5 | GR:0020020 | [LOC_Os05g09480](http://www.gramene.org/Oryza_sativa/geneview?gene=LOC_Os05g09480) | 0 |
| [*iaa17*](http://www.gramene.org/db/genes/search_gene?acc=GR:0020021) | response to auxin stimulus, protein dimerization activity, nucleus, regulation of transcription, regulation of translation | 5 | GR:0020021 | [LOC_Os05g14180](http://www.gramene.org/Oryza_sativa/geneview?gene=LOC_Os05g14180) | 0 |
| [*iaa18*](http://www.gramene.org/db/genes/search_gene?acc=GR:0020022) | response to auxin stimulus, protein dimerization activity, nucleus, regulation of transcription, regulation of translation | 5 | GR:0020022 | [LOC_Os05g44810](http://www.gramene.org/Oryza_sativa/geneview?gene=LOC_Os05g44810) | 0 |
| [*iaa19*](http://www.gramene.org/db/genes/search_gene?acc=GR:0020023) | response to auxin stimulus, protein dimerization activity, nucleus, plastid, regulation of transcription, regulation of translation | 5 | GR:0020023 | [LOC_Os05g48590](http://www.gramene.org/Oryza_sativa/geneview?gene=LOC_Os05g48590) | 0.00367 |
| [*iaa2*](http://www.gramene.org/db/genes/search_gene?acc=GR:0020006) | response to auxin stimulus, intracellular, regulation of translation | 1 | GR:0020006 | [LOC_Os01g09450](http://www.gramene.org/Oryza_sativa/geneview?gene=LOC_Os01g09450) | 0.01958 |
| [*iaa20*](http://www.gramene.org/db/genes/search_gene?acc=GR:0020024) | response to auxin stimulus, protein dimerization activity, nucleus, plastid, regulation of transcription, regulation of translation | 6 | GR:0020024 | [LOC_Os06g07040](http://www.gramene.org/Oryza_sativa/geneview?gene=LOC_Os06g07040) | 0 |
| [*iaa21*](http://www.gramene.org/db/genes/search_gene?acc=GR:0020025) | response to auxin stimulus, protein dimerization activity, nucleus, regulation of transcription, regulation of translation | 6 | GR:0020025 | [LOC_Os06g22870](http://www.gramene.org/Oryza_sativa/geneview?gene=LOC_Os06g22870) | 0 |
| [*iaa23*](http://www.gramene.org/db/genes/search_gene?acc=GR:0020027) | response to auxin stimulus, protein dimerization activity, nucleus, plastid, regulation of transcription, regulation of translation | 6 | GR:0020027 | [LOC_Os06g39590](http://www.gramene.org/Oryza_sativa/geneview?gene=LOC_Os06g39590) | 0 |
| [*iaa26*](http://www.gramene.org/db/genes/search_gene?acc=GR:0020030) | response to auxin stimulus, protein dimerization activity, nucleus, regulation of transcription, regulation of translation | 9 | GR:0020030 | [LOC_Os09g35870](http://www.gramene.org/Oryza_sativa/geneview?gene=LOC_Os09g35870) | 0 |
| [*iaa27*](http://www.gramene.org/db/genes/search_gene?acc=GR:0020031) | response to auxin stimulus, plastid | 11 | GR:0020031 | [LOC_Os11g11410](http://www.gramene.org/Oryza_sativa/geneview?gene=LOC_Os11g11410) | 0.01287 |
| [*iaa28*](http://www.gramene.org/db/genes/search_gene?acc=GR:0020032) | response to auxin stimulus | 11 | GR:0020032 | [LOC_Os11g11420](http://www.gramene.org/Oryza_sativa/geneview?gene=LOC_Os11g11420) | 0 |
| [*iaa28*](http://www.gramene.org/db/genes/search_gene?acc=GR:0020028) | response to auxin stimulus, protein dimerization activity, nucleus, plastid, regulation of transcription, regulation of translation | 7 | GR:0020028 | [LOC_Os07g08460](http://www.gramene.org/Oryza_sativa/geneview?gene=LOC_Os07g08460) | 0.00521 |
| [*iaa29*](http://www.gramene.org/db/genes/search_gene?acc=GR:0020033) | response to auxin stimulus | 11 | GR:0020033 | [LOC_Os11g11430](http://www.gramene.org/Oryza_sativa/geneview?gene=LOC_Os11g11430) | 0 |
| [*iaa3*](http://www.gramene.org/db/genes/search_gene?acc=GR:0020007) | response to auxin stimulus, protein dimerization activity, nucleus, plastid, regulation of transcription, regulation of translation | 1 | GR:0020007 | [LOC_Os01g13030](http://www.gramene.org/Oryza_sativa/geneview?gene=LOC_Os01g13030) | 0 |
| [*iaa30*](http://www.gramene.org/db/genes/search_gene?acc=GR:0020034) | response to auxin stimulus, plastid | 12 | GR:0020034 | [LOC_Os12g40890](http://www.gramene.org/Oryza_sativa/geneview?gene=LOC_Os12g40890) | 0 |
| [*iaa31*](http://www.gramene.org/db/genes/search_gene?acc=GR:0020035) | response to auxin stimulus | 12 | GR:0020035 | [LOC_Os12g40900](http://www.gramene.org/Oryza_sativa/geneview?gene=LOC_Os12g40900) | 0 |
| [*iaa4*](http://www.gramene.org/db/genes/search_gene?acc=GR:0020008) | response to auxin stimulus, mitochondrion | 1 | GR:0020008 | [LOC_Os01g18360](http://www.gramene.org/Oryza_sativa/geneview?gene=LOC_Os01g18360) | 0.00763 |
| [*iaa5*](http://www.gramene.org/db/genes/search_gene?acc=GR:0020009) | response to auxin stimulus, protein dimerization activity, nucleus, plastid, regulation of transcription, regulation of translation | 1 | GR:0020009 | [LOC_Os01g48450](http://www.gramene.org/Oryza_sativa/geneview?gene=LOC_Os01g48450) | 0 |
| [*iaa6*](http://www.gramene.org/db/genes/search_gene?acc=GR:0020010) | response to auxin stimulus, protein dimerization activity, nucleus, regulation of transcription, regulation of translation | 1 | GR:0020010 | [LOC_Os01g53880](http://www.gramene.org/Oryza_sativa/geneview?gene=LOC_Os01g53880) | 0 |
| [*iaa7*](http://www.gramene.org/db/genes/search_gene?acc=GR:0020011) | response to auxin stimulus, protein dimerization activity, nucleus, regulation of transcription, regulation of translation | 2 | GR:0020011 | [LOC_Os02g13520](http://www.gramene.org/Oryza_sativa/geneview?gene=LOC_Os02g13520) | 0 |
| [*iaa8*](http://www.gramene.org/db/genes/search_gene?acc=GR:0020012) | response to auxin stimulus, protein dimerization activity, nucleus, plastid, regulation of transcription, regulation of translation | 2 | GR:0020012 | [LOC_Os02g49160](http://www.gramene.org/Oryza_sativa/geneview?gene=LOC_Os02g49160) | 0.0069 |
| [*iaa9*](http://www.gramene.org/db/genes/search_gene?acc=GR:0020013) | response to auxin stimulus, protein dimerization activity, nucleus, regulation of transcription, regulation of translation | 2 | GR:0020013 | [LOC_Os02g56120](http://www.gramene.org/Oryza_sativa/geneview?gene=LOC_Os02g56120) | 0 |
| [*kao*](http://www.gramene.org/db/genes/search_gene?acc=GR:0020074) | cytoplasmic membrane-bound vesicle, electron transport, gibberellic acid metabolism, monooxygenase activity, secretory pathway | 6 | GR:0020074 | [LOC_Os06g02030](http://www.gramene.org/Oryza_sativa/geneview?gene=LOC_Os06g02030) | 0 |
| [*ks1*](http://www.gramene.org/db/genes/search_gene?acc=GR:0020067) | metabolism, gibberellic acid metabolism, plastid | 4 | GR:0020067 | [LOC_Os04g52230](http://www.gramene.org/Oryza_sativa/geneview?gene=LOC_Os04g52230) | 0.00788 |
| [*ks2*](http://www.gramene.org/db/genes/search_gene?acc=GR:0020068) | lyase activity, metabolism, gibberellic acid metabolism | 4 | GR:0020068 | [LOC_Os04g52240](http://www.gramene.org/Oryza_sativa/geneview?gene=LOC_Os04g52240) | 0.00127 |
| [*ks4*](http://www.gramene.org/db/genes/search_gene?acc=GR:0020070) | lyase activity, metabolism, gibberellic acid metabolism, plastid | 4 | GR:0020070 | [LOC_Os04g10060](http://www.gramene.org/Oryza_sativa/geneview?gene=LOC_Os04g10060) | 0.00264 |
| [*lrd13*](http://www.gramene.org/db/genes/search_gene?acc=GR:0060908) | biological process unknown, transcription factor activity, nucleus, regulation of transcription, DNA-dependent, DNA binding | 5 | GR:0060908 | [LOC_Os05g45410](http://www.gramene.org/Oryza_sativa/geneview?gene=LOC_Os05g45410) | 0 |
| [*Msp1*](http://www.gramene.org/db/genes/search_gene?acc=GR:0061178) | protein-tyrosine kinase activity, protein serine/threonine kinase activity, megasporogenesis, ATP binding, tapetal layer development, cytoplasmic membrane-bound vesicle, protein amino acid phosphorylation, microsporogenesis, membrane, secretory pathway | 1 | GR:0061178 | [LOC_Os01g68870](http://www.gramene.org/Oryza_sativa/geneview?gene=LOC_Os01g68870) | 0.00099 |
| [*Pair1*](http://www.gramene.org/db/genes/search_gene?acc=GR:0100119) | meiotic anaphase I, meiotic telophase I, microsporogenesis, megasporogenesis, nucleus, meiotic prophase I | 3 | GR:0100119 | [LOC_Os03g01590](http://www.gramene.org/Oryza_sativa/geneview?gene=LOC_Os03g01590) | 0 |
| [*Pair2*](http://www.gramene.org/db/genes/search_gene?acc=GR:0100120) | nuclear chromosome, synapsis, zygotene, microsporogenesis, megasporogenesis, nucleus, leptotene | 9 | GR:0100120 | [LOC_Os09g32930](http://www.gramene.org/Oryza_sativa/geneview?gene=LOC_Os09g32930) | 0.00327 |
| [*Phya*](http://www.gramene.org/db/genes/search_gene?acc=GR:0060611) | mitochondrion, two-component sensor activity, regulation of transcription, DNA-dependent, protein histidine kinase activity, G-protein coupled photoreceptor activity, ATP binding, signal transducer activity, signal transduction, membrane | 3 | GR:0060611 | [LOC_Os03g51030](http://www.gramene.org/Oryza_sativa/geneview?gene=LOC_Os03g51030) | 0 |
| [*Pita*](http://www.gramene.org/db/genes/search_gene?acc=GR:0060627) | response to fungi, plastid, defense response, apoptosis, ATP binding | 12 | GR:0060627 | [LOC_Os12g18360](http://www.gramene.org/Oryza_sativa/geneview?gene=LOC_Os12g18360) | 0.00093 |
| [*plt1*](http://www.gramene.org/db/genes/search_gene?acc=GR:0061116) |  | 10 | GR:0061116 | [LOC_Os10g26340](http://www.gramene.org/Oryza_sativa/geneview?gene=LOC_Os10g26340) | 0 |
| [*Q67W57*](http://www.gramene.org/db/genes/search_gene?acc=GR:0100192) | hydrogen-transporting ATP synthase activity, rotational mechanism, proton-transporting ATP synthase, catalytic core (sensu Eukaryota), hydrogen-transporting ATPase activity, rotational mechanism, mitochondrion, proton-transporting two-sector ATPase complex, ATP synthesis coupled proton transport, ATP biosynthesis | 6 | GR:0100192 | [LOC_Os06g43850](http://www.gramene.org/Oryza_sativa/geneview?gene=LOC_Os06g43850) | 0 |
| [*Q6YW12*](http://www.gramene.org/db/genes/search_gene?acc=GR:0100194) | hydrogen-transporting ATP synthase activity, rotational mechanism, proton-transporting ATP synthase, catalytic core (sensu Eukaryota), hydrogen-transporting ATPase activity, rotational mechanism, mitochondrion, proton-transporting two-sector ATPase complex, ATP synthesis coupled proton transport, ATP biosynthesis | 8 | GR:0100194 | [LOC_Os08g15170](http://www.gramene.org/Oryza_sativa/geneview?gene=LOC_Os08g15170) | 0 |
| [*Q8GTK7*](http://www.gramene.org/db/genes/search_gene?acc=GR:0100193) | hydrogen-transporting ATP synthase activity, rotational mechanism, proton-transporting ATP synthase, catalytic core (sensu Eukaryota), mitochondrion, proton transport, ATP synthesis coupled proton transport, ATP biosynthesis | 7 | GR:0100193 | [LOC_Os07g31300](http://www.gramene.org/Oryza_sativa/geneview?gene=LOC_Os07g31300) | 0 |
| [*qcr6*](http://www.gramene.org/db/genes/search_gene?acc=GR:0100187) | mitochondrion, respiratory chain complex III (sensu Eukaryota), mitochondrial electron transport, ubiquinol to cytochrome c, electron transporter, transferring electrons within CoQH2-cytochrome c reductase complex activity | 11 | GR:0100187 | [LOC_Os11g06340](http://www.gramene.org/Oryza_sativa/geneview?gene=LOC_Os11g06340) | 0 |
| [*qcr7*](http://www.gramene.org/db/genes/search_gene?acc=GR:0100188) | mitochondrion, electron transport, ubiquinol-cytochrome-c reductase activity, respiratory chain complex III (sensu Eukaryota), mitochondrial electron transport, ubiquinol to cytochrome c, electron transporter, transferring electrons within CoQH2-cytochrome c reductase complex activity | 3 | GR:0100188 | [LOC_Os03g59220](http://www.gramene.org/Oryza_sativa/geneview?gene=LOC_Os03g59220) | 0 |
| [*Ra*](http://www.gramene.org/db/genes/search_gene?acc=GR:0060719) | mitochondrion, plastid, flavonoid metabolism | 4 | GR:0060719 | [LOC_Os04g47080](http://www.gramene.org/Oryza_sativa/geneview?gene=LOC_Os04g47080) | 0.00184 |
| [*Rb*](http://www.gramene.org/db/genes/search_gene?acc=GR:0060720) | flavonoid metabolism | 1 | GR:0060720 | [LOC_Os01g39580](http://www.gramene.org/Oryza_sativa/geneview?gene=LOC_Os01g39580) | 0 |
| [*Rcn1*](http://www.gramene.org/db/genes/search_gene?acc=GR:0100117) | phosphatidylethanolamine binding, inflorescence development, regulation of timing of transition from vegetative to reproductive phase | 11 | GR:0100117 | [LOC_Os11g05470](http://www.gramene.org/Oryza_sativa/geneview?gene=LOC_Os11g05470) | 0.00663 |
| [*Rcn3*](http://www.gramene.org/db/genes/search_gene?acc=GR:0100116) | phosphatidylethanolamine binding, plastid | 12 | GR:0100116 | [LOC_Os12g05590](http://www.gramene.org/Oryza_sativa/geneview?gene=LOC_Os12g05590) | 0 |
| [*rr1*](http://www.gramene.org/db/genes/search_gene?acc=GR:0020055) | two-component response regulator activity, regulation of transcription, DNA-dependent, response to cytokinin stimulus, two-component signal transduction system (phosphorelay) | 1 | GR:0020055 | [LOC_Os04g36070](http://www.gramene.org/Oryza_sativa/geneview?gene=LOC_Os04g36070) | 0.00492 |
| [*rr2*](http://www.gramene.org/db/genes/search_gene?acc=GR:0020056) | two-component response regulator activity, regulation of transcription, DNA-dependent, response to cytokinin stimulus, two-component signal transduction system (phosphorelay) | 2 | GR:0020056 | [LOC_Os02g35180](http://www.gramene.org/Oryza_sativa/geneview?gene=LOC_Os02g35180) | 0.02122 |
| [*rr3*](http://www.gramene.org/db/genes/search_gene?acc=GR:0020057) | two-component response regulator activity, plastid, regulation of transcription, DNA-dependent, response to cytokinin stimulus, two-component signal transduction system (phosphorelay) | 2 | GR:0020057 | [LOC_Os02g58350](http://www.gramene.org/Oryza_sativa/geneview?gene=LOC_Os02g58350) | 0 |
| [*rr4*](http://www.gramene.org/db/genes/search_gene?acc=GR:0020058) | two-component response regulator activity, regulation of transcription, DNA-dependent, response to cytokinin stimulus, two-component signal transduction system (phosphorelay) | 1 | GR:0020058 | [LOC_Os01g72330](http://www.gramene.org/Oryza_sativa/geneview?gene=LOC_Os01g72330) | 0 |
| [*rr5*](http://www.gramene.org/db/genes/search_gene?acc=GR:0020059) | mitochondrion, two-component response regulator activity, regulation of transcription, DNA-dependent, response to cytokinin stimulus, two-component signal transduction system (phosphorelay) | 4 | GR:0020059 | [LOC_Os04g44280](http://www.gramene.org/Oryza_sativa/geneview?gene=LOC_Os04g44280) | 0 |
| [*rr6*](http://www.gramene.org/db/genes/search_gene?acc=GR:0020060) | mitochondrion, two-component response regulator activity, plastid, regulation of transcription, DNA-dependent, response to cytokinin stimulus, two-component signal transduction system (phosphorelay) | 4 | GR:0020060 | [LOC_Os04g57720](http://www.gramene.org/Oryza_sativa/geneview?gene=LOC_Os04g57720) | 0.00577 |
| [*rr7*](http://www.gramene.org/db/genes/search_gene?acc=GR:0020061) | two-component response regulator activity, regulation of transcription, DNA-dependent, response to cytokinin stimulus, two-component signal transduction system (phosphorelay) | 7 | GR:0020061 | [LOC_Os07g26720](http://www.tigr.org/tigr-scripts/euk_manatee/BacAnnotationPage.cgi?db=osa1&asmbl_id=11977) | 0.06374 |
| [*rr8*](http://www.gramene.org/db/genes/search_gene?acc=GR:0020062) | two-component response regulator activity, regulation of transcription, DNA-dependent, response to cytokinin stimulus, two-component signal transduction system (phosphorelay) | 8 | GR:0020062 | [LOC_Os08g28950](http://www.gramene.org/Oryza_sativa/geneview?gene=LOC_Os08g28950) | 0 |
| [*rr9*](http://www.gramene.org/db/genes/search_gene?acc=GR:0020063) | two-component response regulator activity, plastid, regulation of transcription, DNA-dependent, response to cytokinin stimulus, two-component signal transduction system (phosphorelay) | 12 | GR:0020063 | [LOC_Os12g04500](http://www.gramene.org/Oryza_sativa/geneview?gene=LOC_Os12g04500) | 0 |
| [*rugt-5*](http://www.gramene.org/db/genes/search_gene?acc=GR:0020036) | transferase activity, transferring hexosyl groups, cytoplasmic membrane-bound vesicle, UDP-glycosyltransferase activity, metabolism, secretory pathway | 1 | GR:0020036 | [LOC_Os01g41430](http://www.gramene.org/Oryza_sativa/geneview?gene=LOC_Os01g41430) | 0 |
| [*sd1*](http://www.gramene.org/db/genes/search_gene?acc=GR:0060842) | gibberellic acid biosynthesis, gibberellin 20-oxidase activity, unidimensional cell growth, response to abiotic stimulus | 1 | GR:0060842 | [LOC_Os01g66100](http://www.gramene.org/Oryza_sativa/geneview?gene=LOC_Os01g66100) | 0.00412 |
| [*sdhA*](http://www.gramene.org/db/genes/search_gene?acc=GR:0100183) | oxidoreductase activity, acting on the CH-CH group of donors, mitochondrial electron transport, succinate to ubiquinone, mitochondrion, respiratory chain complex II (sensu Eukaryota), disulfide oxidoreductase activity, oxidoreductase activity, electron transport, FAD binding, tricarboxylic acid cycle, succinate dehydrogenase (ubiquinone) activity, succinate dehydrogenase activity | 7 | GR:0100183 | [LOC_Os07g04240](http://www.gramene.org/Oryza_sativa/geneview?gene=LOC_Os07g04240) | 0 |
| [*sdhB*](http://www.gramene.org/db/genes/search_gene?acc=GR:0100184) | electron carrier activity, mitochondrion, respiratory chain complex II (sensu Eukaryota), oxidoreductase activity, electron transport, tricarboxylic acid cycle, iron ion binding, succinate dehydrogenase (ubiquinone) activity, succinate dehydrogenase activity | 9 | GR:0100184 | [LOC_Os09g20440](http://www.gramene.org/Oryza_sativa/geneview?gene=LOC_Os09g20440) | 0.01495 |
| [*Sk1*](http://www.gramene.org/db/genes/search_gene?acc=GR:0100111) | shikimate kinase activity, aromatic amino acid family biosynthesis, plastid | 2 | GR:0100111 | [LOC_Os02g51410](http://www.gramene.org/Oryza_sativa/geneview?gene=LOC_Os02g51410) | 0 |
| [*Sk2*](http://www.gramene.org/db/genes/search_gene?acc=GR:0100112) | shikimate kinase activity, aromatic amino acid family biosynthesis, plastid | 6 | GR:0100112 | [LOC_Os06g12150](http://www.gramene.org/Oryza_sativa/geneview?gene=LOC_Os06g12150) | 0 |
| [*Sk3*](http://www.gramene.org/db/genes/search_gene?acc=GR:0100113) | shikimate kinase activity, aromatic amino acid family biosynthesis, plastid, ATP binding | 4 | GR:0100113 | [LOC_Os04g54800](http://www.gramene.org/Oryza_sativa/geneview?gene=LOC_Os04g54800) | 0 |
| [*slr*](http://www.gramene.org/db/genes/search_gene?acc=GR:0060897) | regulation of gene expression, epigenetic | 3 | GR:0060897 | [LOC_Os03g49990](http://www.gramene.org/Oryza_sativa/geneview?gene=LOC_Os03g49990) | 0.00331 |
| [*wx*](http://www.gramene.org/db/genes/search_gene?acc=GR:0061010) | amyloplast, amyloplast starch grain, starch metabolism, plastid, glycogen (starch) synthase activity | 6 | GR:0061010 | [LOC_Os06g04200](http://www.gramene.org/Oryza_sativa/geneview?gene=LOC_Os06g04200) | 0 |
| [*Xa1*](http://www.gramene.org/db/genes/search_gene?acc=GR:0061011) | defense response to pathogen, DNA binding, apoptosis, ATP binding | 4 | GR:0061011 | [LOC_Os04g53120](http://www.gramene.org/Oryza_sativa/geneview?gene=LOC_Os04g53120) | 0.08145 |
| [*Xa21*](http://www.gramene.org/db/genes/search_gene?acc=GR:0061029) | cytoplasmic membrane-bound vesicle, protein amino acid phosphorylation, protein serine/threonine kinase activity, protein kinase activity, secretory pathway, ATP binding | 11 | GR:0061029 | [LOC_Os11g35500](http://www.gramene.org/Oryza_sativa/geneview?gene=LOC_Os11g35500) | 0.02557 |
| [*xa5*](http://www.gramene.org/db/genes/search_gene?acc=GR:0061015) | transcription initiation from RNA polymerase II promoter, defense response to pathogenic bacteria, RNA polymerase II transcription factor activity, transcription factor TFIIA complex | 5 | GR:0061015 | [LOC_Os05g01710](http://www.gramene.org/Oryza_sativa/geneview?gene=LOC_Os05g01710) | 0 |
| [*Xip*](http://www.gramene.org/db/genes/search_gene?acc=GR:0100115) | hydrolase activity, enzyme inhibitor activity, cytoplasmic membrane-bound vesicle, secretory pathway | 11 | GR:0100115 | [LOC_Os11g47580](http://www.gramene.org/Oryza_sativa/geneview?gene=LOC_Os11g47580) | 0.01006 |
