# Supplementary material for: Genomic Variation in Rice: Genesis of Highly Polymorphic Linkage Blocks during Domestication
Source: PLoS Genet. 2006 Nov 17;2(11):e199. doi: 10.1371/journal.pgen.0020199 (PMC1636696; doi:10.1371/journal.pgen.0020199)
Supplement: Supplementary file 9 [file pgen.0020199.st008.doc]

**Table S8.** Statistics for 10 reference genes in rice cultivars and *O. rufipogon*, the average of which is also presented in Table 1. π_silent_ is the number of synonymous changes per site averaged over all pairwise comparisons between sequences, *D_T_* is Tajima’s D statistic , and *r^2^* , the squared allele-frequency correlation, is a common measure of LD.

| Accession number | Gene | Chr | No. sites | π_silent_×10^3^ | | | |  | *D_T_* | | | |  | *r^2^* | | | |
| --- | --- | --- | --- | --- | --- | --- | --- | --- | --- | --- | --- | --- | --- | --- | --- | --- | --- |
|  |  |  |  | J | I | OS | OR |  | J | I | OS | OR |  | J | I | OS | OR |
| AJ133787 | GIGANTEA | 1 | 981 | 5.51 | 14.81 | 4.05 | 5.99 |  | 0.362 | 0.71 | -1.734 | -2.083^*^ |  | 0.593 | 0.230 | 0.202 | 0.082 |
| AF326768 | *EMF1* | 1 | 915 | 0.66 | 1.64 | 3.96 | 7.15 |  | -1.562 | -0.072 | 1.561 | -1.310 |  | 1.000 | 0.214 | 0.251 | 0.360 |
| AB071330 | *qSH-1* | 1 | 571 | 0.58 | 1.33 | 2.08 | 3.67 |  | -1.451 | 0.628 | 1.610 | -0.528 |  | 1.000 | 0.091 | 0.245 | 0.471 |
| AB103395 | *MSP1* | 1 | 959 | 0.49 | 0.52 | 0.36 | 7.4 |  | -0.850 | 1.212 | 0.409 | -1.206 |  | 0.455 | na | 0.055 | 0.652 |
| AB050095 | *Spl7* | 5 | 899 | 0.19 | 0.30 | 0 | 9.87 |  | -1.141 | -1.491 | 2.269^*^ | -1.312 |  | na | 0.005 | 0.002 | 0.550 |
| AB061311 | *HKT1* | 6 | 775 | 0.22 | 2.08 | 7.58 | 7.1 |  | -1.141 | 0.009 | 0.582 | -0.524 |  | na | 0.198 | 0.228 | 0.052 |
| D10752 | *sbe1* | 6 | 704 | 0.43 | 0.52 | 7.88 | 6.4 |  | -0.195 | 0.324 | -0.818 | 0.105 |  | na | na | na | 0.013 |
| AY452478 | *COC1* | 8 | 937 | 1.05 | 1.18 | 4.56 | 6.64 |  | -0.902 | 0.540 | 1.277 | -1.869^*^ |  | 0.591 | 0.101 | 0.402 | 0.149 |
| U37133 | *Xa21* | 11 | 1088 | 1.69 | 3.52 | 1.24 | 1.32 |  | -2.067^**^ | 0.932 | -2.414^**^ | -2.162^**^ |  | 0.820 | 0.412 | 0.291 | 0.266 |
| AY196754 | *Pi-ta* | 12 | 1054 | 0.16 | 1.52 | 3.29 | 2.77 |  | -1.141 | -2.207^**^ | 0.247 | -1.165 |  | na | 0.548 | 0.462 | 0.751 |
| Average |  |  | 888.3 | 1.10 | 2.74 | 3.50 | 5.83 |  | -1.009 | 0.059 | 0.299 | -1.205 |  | 0.743 | 0.225 | 0.237 | 0.335 |

Chr. – chromosome; J – *O. sativa* ssp. *japonica*; I– *O. sativa* ssp. *indica*; OS– *O. sativa,* including japonicas and indicas; OR – *O. rufipogon*; na – not available.

^*^ *P*<0.05, ^**^ *P*<0.01
